# Elucidating the Photocatalytic Behavior of TiO_2_-SnS_2_ Composites Based on Their Energy Band Structure

**DOI:** 10.3390/ma11061041

**Published:** 2018-06-19

**Authors:** Marin Kovacic, Jozefina Katic, Hrvoje Kusic, Ana Loncaric Bozic, Mirjana Metikos Hukovic

**Affiliations:** Faculty of Chemical Engineering and Technology, University of Zagreb, Marulicev trg 19, Zagreb 10000, Croatia; mkovacic1@fkit.hr (M.K.); jkatic@fkit.hr (J.K.); abozic@fkit.hr (A.L.B.)

**Keywords:** TiO_2_-SnS_2_ composite catalysts, semiconducting parameters, energy band diagram construction, solar photocatalytic water treatment, diclofenac

## Abstract

TiO_2_-SnS_2_ composite semiconducting photocatalysts with different building component ratios were prepared by hydrothermal synthesis (TiO_2_-SnS_2_-HT) and by immobilization of commercial TiO_2_ and SnS_2_ particles (TiO_2_-SnS_2_-COMM). The band gap values, which determine the catalysts’ photoactivity, were examined by diffuse reflectance spectroscopy and Kubelka–Munk transformations. The catalysts’ surface properties: specific surface area, charge and adsorption capacitance at the solid–solution interface were characterized using BET analysis, potentiometric titration and electrochemical impedance spectroscopy, respectively. The electronic band structure of TiO_2_-SnS_2_ photocatalyst, as the key property for the solar-driven photocatalysis, was deduced from the thermodynamic data and the semiconducting parameters (type of semiconductivity, concentration of the charge carriers, flat band potential) obtained by Mott–Schottky analysis. The photoactivity of both composites was studied in photocatalytic treatment of diclofenac (DCF) under simulated solar irradiation and was compared to the benchmark photocatalyst (TiO_2_ P25) activity. The influence of process parameters, such as pH, H_2_O_2_, and composite formulation on the effectiveness of DCF removal and conversion was investigated and discussed by employing response surface modeling (RSM) approach. The photocatalytic efficiency of both composite materials was discussed on the basis of the hetereojunction formation that facilitated the photoelectron transfer, promoting more efficient photocatalytic degradation of DCF.

## 1. Introduction

Semiconductor materials have become of great significance owing to their wide potential applications in important fields of modern society, such as electrochemical sensors, organic synthesis, self-cleaning surfaces, solar driven hydrogen production, and wastewater treatment [1,2,3,4,5]. Current wastewater treatment technologies can be assisted by semiconductor photocatalysis in overcoming inadequate removal of emerging contaminants, including pharmaceuticals [5,6,7].

TiO_2_ is de facto the gold standard in photocatalytic wastewater treatment, due to its low cost, chemical stability, high activity, and low toxicity. A widespread and cost-effective application of TiO_2_ is hindered by its prohibitively wide band gap (*E*_g_ ≈ 3.0−3.2 eV), limiting photoactivation only to UV part of the solar spectrum [8]. Transition metal sulfides, on the other hand, have significantly narrow band gaps (*E*_g_ < 2 eV) [9,10,11] and are photoactive under visible light. An increase in cost-effectiveness from narrow band gaps is easy to comprehend, considering that only 5% of solar irradiation belongs to UV [12]. A major hurdle for broader sulfide photocatalyst application is their tendency towards photocorrosion [13,14].

The combination of different semiconductor materials may have complementary properties and create new materials with optimal performances in visible-light-driven photocatalyst water treatment systems. For instance, forming unique semiconductor–semiconductor junctions, depending on the semiconductors’ band-structure, can extend the spectral range for light absorption and enhance electron–hole separation [15]. A typical heterojunction photocatalyst is CdS-TiO_2_ composite, which can be activated by visible light owing to the narrow band gap of CdS component. Composite catalyst exhibits high quantum efficiency since the photoelectrons generated in CdS can easily migrate into TiO_2_, leading to the depression of photoelectron–hole recombination in TiO_2_ [16]. However, CdS-TiO_2_ composite displays poor durability due to CdS photocorrosion [17,18], whilst also leaching toxic Cd^2+^ ions, thereby causing adverse effects in water treatment purposes [19]. The SnS_2_ semiconductor is considered to be a viable option, due to its narrow band gap, low toxicity, and better photostability in comparison to CdS [4,20,21].

The aim of this study was to determine the properties of TiO_2_-SnS_2_ composite materials prepared by different methods. Barrier properties (electronic conductivity, corrosion resistance) and electronic structure (semiconducting properties) of TiO_2_-SnS_2_ composites were investigated in situ using electrochemical impedance spectroscopy (EIS) and Mott–Schottky analysis [22,23,24]. Thermodynamic stability of TiO_2_-SnS_2_ hybrid systems against cathodic and anodic decomposition was discussed. Within this study, the surface and optical properties of the composites were also characterized using BET method (specific surface area), potentiometric titration (point of zero charge, pH_PZC_), and diffuse reflectance spectroscopy (DRS) (light absorption capacity and corresponding band gap energy calculation). The activity of TiO_2_-SnS_2_ composites under solar irradiation was studied towards diclofenac (DCF) removal and conversion. DCF is one of the most commonly used analgesics, recently included in the “watch list” of priority substances in the EU Water Framework Directive [25]. Its presence in natural recipients due to inadequate removal by current wastewater treatment technologies, and the potential to cause adverse effects in aquatic ecosystems is documented [26,27]. The DCF removal and conversion extents during solar-driven treatment using TiO_2_-SnS_2_ composites were evaluated in correlation with process parameters studied: initial pH, composite formulation, and concentration of oxidant (H_2_O_2_) using a statistical/empirical approach employing response surface modeling (RSM). The obtained results were correlated with determined surface and semiconducting properties of applied photocatalytic composite materials.

## 2. Materials and Methods

### 2.1. Chemicals

Titanium (IV) butoxide (TBO) (97%, Acros Organics, Geel, Belgium), tin (IV) chloride (98%, Sigma-Aldrich, Taufkirchen, Germany ), thioacetamide (≥99.0%, Sigma-Aldrich, Taufkirchen, Germany), ethanol (abs., Gram-mol, Zagreb, Croatia), and acetic acid (≥99.7%, Carlo Erba Reagents, Val-de-Reuil, France), were used in synthesis of TiO_2_, SnS_2_ and TiO_2_-SnS_2_ composite catalysts, denoted as TiO_2_-SnS_2_-HT. Commercial TiO_2_ (AEROXIDE P25, Evonik, Essen, Germany) and SnS_2_ particles (MKN-900, MKnano, MK Impex Corp., Ontario, Canada) were used for the preparation of composite denoted as TiO_2_-SnS_2_-COMM. Titanium tetraisopropoxide (TTIP) (97%, Sigma-Aldrich), ethanol, perchloric acid (70%, Kemika, Zagreb, Croatia), tetraethyl orthosilicate (TEOS) (99% GC grade, Sigma-Aldrich), hydrochloric acid (36.5%, Gram-mol, Zagreb, Croatia), and Levasil^®^ 200/30 (Obermeier, Bad-Berleburg, Germany) were used for immobilization of thin films. Spectroscopically pure titanium foil (Ti, 99.9%, Alfa Aesar, Karlsruhe, Germany) was used as the substrate for working electrodes in electrochemical measurements, while electrolyte contained sodium chloride (p.a., Kemika, Zagreb, Croatia). Diclofenac sodium salt (DCF) (p.a., Sigma-Aldrich, Taufkirchen, Germany) was used as a model water pollutant. Following auxiliary chemicals were used as well: hydrogen peroxide (*w* = 30%, Gram-mol, Zagreb, Croatia), sodium hydroxide (p.a., Kemika, Zagreb, Croatia), sulfuric acid (>96%, Kemika, Zagreb, Croatia), ammonium metavanadate (p.a., Kemika, Zagreb, Croatia), methanol (HPLC grade, Sigma-Aldrich, Taufkirchen, Germany), orthophosphoric acid (*w* ≈ 85%, Fluka, Bucharest, Romania), potassium nitrate (p.a., Kemika, Zagreb, Croatia), potassium hydroxide (p.a., Kemika, Zagreb, Croatia), and nitric acid (≥90%, Sigma Aldrich, Taufkirchen, Germany).

### 2.2. Photocatalyst Synthesis and Immobilization

Hydrothermal method, according to procedure adopted from Zhang et al. [9], was applied to prepare TiO_2_, SnS_2_ and their composites denoted as TiO_2_-SnS_2_-HT. TiO_2_ was synthesized by dissolving an aliquot of TBO precursor in a 5% *v*/*v* solution of acetic acid in ethanol with a constant stirring in Teflon reaction vessel, which was then transferred to a stainless steel autoclave, where treated for 12 h at 180 °C. After cooling naturally to room temperature, the obtained suspension was rinsed with distilled water, centrifuged (3500 rpm for 3 min), dried in a vacuum (3 h at 60 °C), and then homogenized with a porcelain pestle and mortar. The same procedure was applied for SnS_2_ and TiO_2_-SnS_2_-HT composite synthesis, using corresponding precursors (tin(IV) chloride, thioacetamide, and TBO in later case), while their stoichiometric were varied to obtain the composite with different SnS_2_ content (wt % of 5, 27.5, 50, 72.5). Composites denoted as TiO_2_-SnS_2_-COMM were prepared from the commercial TiO_2_ and SnS_2_ particles during immobilization procedure, whereas their weights were varied to obtain abovementioned ratios.

The immobilization procedure included the addition of as-prepared composite or a mixture of commercial particles into titania/silica binder sol (TSB) to obtain TiO_2_-SnS_2_-HT and TiO_2_-SnS_2_-COMM, respectively. TSB was made by mixing two as-prepared sols (nanocrystaline titania sol synthetized from TTIP and silica sol synthetized from TEOS), colloidal SiO_2_ and ethanol [28]. The suspended particles within TSB sol were firstly homogenized using ultrasonic bath, and then the final sol suspension was immobilized on (i) round titanium discs (*d =* 1 mm, *r* *=* 12 mm) in three layers, and (ii) round glass plates (*r* *=* 35.5 mm) in one layer using spin coating (1500 rpm) technique with KW-4A Spin Coater (Chemat Technology, Northridge, CA, USA). The final fixation step was performed by heating the coated titanium discs or glass plates in a laboratory oven (2 h at 200 °C).

### 2.3. Photocatalyst Characterization

Diffuse reflectance spectra (DRS) of immobilized photocatalysts, were measured using UV–vis spectrophotometer equipped with an integrating sphere, Lambda 650S (Perkin Elmer, Waltham, MA, USA). The acquired reflectance vs. wavelength spectra were transformed into the Kubelka–Munk function (KM) vs. photon energy (*hν*) in order to obtain the band gap values [29,30,31]. Nitrogen adsorption analysis, performed using Gemini 2380 instrument (Micromeritics, Norcross, GA, USA), was used to determine the specific surface area of composites and their pure components. Brunauer–Emmett–Teller (BET) surface area was calculated from BET plot. The samples of immobilized composites were obtained by carefully scratching the films and collecting the corresponding powder, which was then grounded in an agate mortar. The same procedure for obtaining such powdered samples of composites was applied prior determination of point of zero charge (pH_PCZ_) values. Handylab pH/LF portable pH meter (Schott Instruments GmbH, Mainz, Germany) was used for pH monitoring during potentiometric titration applied for the determination of pH_PCZ_ of TiO_2_-SnS_2_ composites. The pH_PCZ_ values were determined according to the modified procedure of Uppal et al. [32], while detail description of applied methodology is provided in the previous study [33].

Electrochemical characterization of photocatalysts immobilized on titanium substrates was performed using Solartron potentiostat/galvanostat 1287 with FRA 1260 in a conventional three-electrode cell: Ti-coated disc was working electrode, the counter electrode was Pt and the reference electrode was Ag|AgCl (*E* *=* 0.208 V vs. SHE). The electrolyte was 3% NaCl solution, pure or spiked with 0.1 mM DCF. The structure of catalyst films–electrolyte interface was investigated at the open circuit potential (*E*_ocp_) using electrochemical impedance spectroscopy (EIS); frequency ranged from 100 kHz to 5 mHz at an *ac* voltage amplitude of ±5 mV. The experimental data were fitted using the complex non-linear least squares fit analysis software [34] and values of elements of proposed electric equivalent circuit (EEC) were derived with χ^2^ values <5 × 10^−3^. The electronic-semiconducting properties of catalyst films were investigated by Mott–Schottky analysis [22,23,24]. The interfacial capacitance values were obtained from EIS measurements as a function of both frequency and applied potential in a rapid polarization scan. Detailed description is given in Supplementary Material.

### 2.4. Photocatalytic Activity under Solar Irradiation

The photocatalytic experiments were performed in a water-jacket batch reactor (*V* *=* 0.09 L and *T* *=* 25.0 ± 0.2 °C). The source of simulated solar irradiation was a 450 W Xenon arc lamp (Osram, Munich, Germany) situated in Oriel/Newport, USA housing with collimating optics. An Oriel AM1.5 G air mass filter was situated in the path of the collimated beam, mimicking solar spectral characteristics when the Sun is at a zenith angle of 48.2°. The light intensity, 124.78 ± 0.11 mW cm^−2^, was determined by pyranometer CMP21 (Kipp & Zonen, Delft, The Netherlands), while the intensity of UV-A irradiation emitted by solar simulator was found to be 2.05 ± 0.07 mW cm^−2^, determined using UVX radiometer equipped with UVX-36 longwave sensor (both UVP, Cambridge, UK). Aqueous solution of DCF (*c*_0_ *=* 0.1 mM) was treated by solar driven photocatalytic processes using TiO_2_-SnS_2_-COMM or TiO_2_-SnS_2_-HT in the presence and absence of H_2_O_2,_ whereas TiO_2_ P25 was used as a benchmark material. The DCF solution with adjusted pH was then spiked with H_2_O_2_ (where applicable) according to full factorial (FFD) or Box–Behnken designs (BBD) (Table 1 and Tables S1–S4, Supplementary Material), which was followed by the immersion of the glass plates into the reactor placed on an orbital shaker DOS-20 (90 rpm, neoLab, Heidelberg, Germany). Adsorption equilibrium was reached within 30 min in a dark, and thereafter the reaction solution was exposed to simulated solar irradiation. The samples (500 μL aliquots) were taken during experiments at −30 (30 min prior to irradiation), 0 (the start of irradiation), 15, 30, 45, and 60 min, filtered using Chromafil XTRA RC (25 mm, 0.45 μm, Macherey Nagel, Düren, Germany), quenched with CH_3_OH and submitted to HPLC analysis. All experiments were repeated at least three times and averages are reported; the reproducibility was >97.3%. DCF concentration was monitored by HPLC analysis, using Series 10 apparatus equipped with UV-DAD, SPD-M10A_VP_ (Shimadzu, Japan), Nucleosil C18 column (5 μm, 25.0 cm × 4.6 mm, Macherey Nagel, Düren, Germany), and mobile phase CH_3_OH 0.1% formic acid operating at 1.0 mL min^−1^ flow. UV–vis spectrophotometer, Lambda EZ 201, Perkin Elmer (USA) was used for the monitoring of H_2_O_2_ by metavanadate method [35]. In desorption tests, the plates used in photocatalytic experiments were immersed in NaOH solution (90 mL, pH 8.00 ± 0.05) and placed on the shaker for 30 min. The solution was analyzed according to the above procedure. The influence of initial pH, H_2_O_2_, and SnS_2_ wt % within TiO_2_-SnS_2_ composites, on DCF removal and conversion by solar driven photocatalytic treatment was correlated by means of RSM. Related calculations, procedure, and analysis are described in Supplementary Material.

## 3. Results and Discussion

### 3.1. Semiconducting and Surface Properties of TiO_2_-SnS_2_ Composite Materials

**Figure 1:**
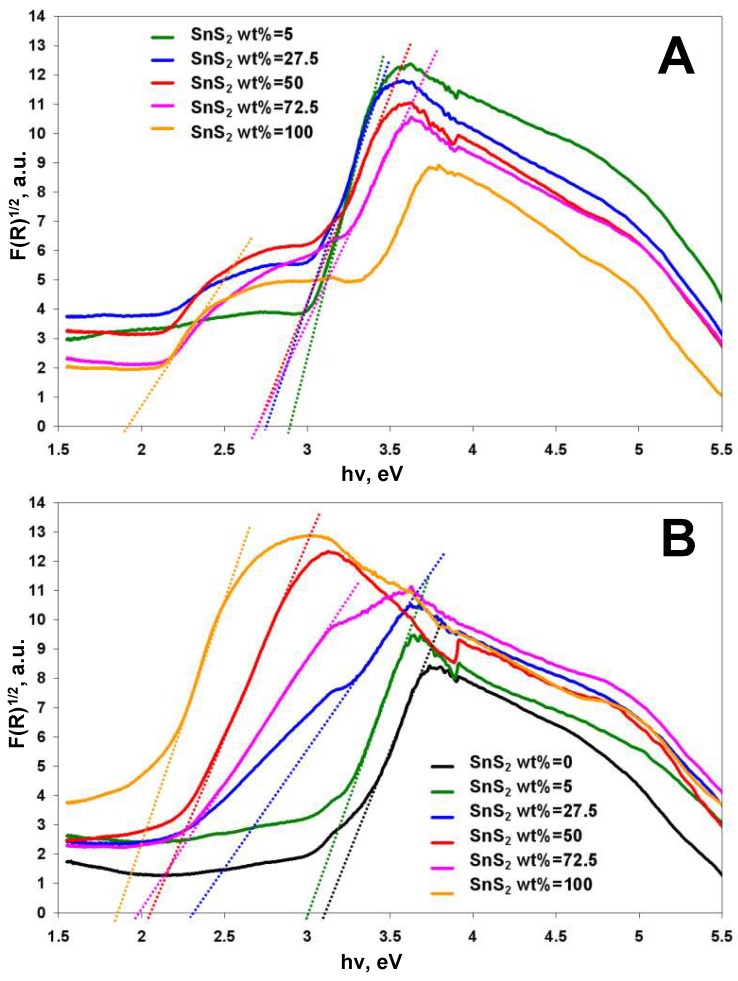
Plot of transformed Kubelka–Munk function vs. the light energy for immobilized TiO_2_-SnS_2_ composites with different SnS_2_ wt %: commercial (COMM) (**A**) and hydrothermal (HT) (**B**).

DRS analysis of TiO_2_-SnS_2_-COMM and TiO_2_-SnS_2_-HT composites, with different SnS_2_ content ranging from 5 to 72.5 wt %, was performed to determine the band gap values, *E*_g_ of composite materials studied. DRS spectra of the pure components TiO_2_-HT, SnS_2_-HT and SnS_2_ MKN-900 were also recorded. The spectra obtained (Figure S1A,B, Supplementary Material) were transformed using Kubelka–Munk function, Figure 1A,B. The *E*_g_ values determined are given in Table 2. While *F*(*R*)^1/2^ vs. *hν* plots of TiO_2_-SnS_2_-HT are characterized with only one flat section, the existence of two flat sections in plots of TiO_2_-SnS_2_-COMM, more emphasized with SnS_2_ wt % increase, point to the segregation of TiO_2_ and SnS_2_ composite phases. Such results are in accordance with the established morphology revealed by SEM/EDX analysis showing phase separation in the COMM composite while the HT composite consisted of TiO_2_ matrix with embedded and homogenously dispersed SnS_2_ small proto-platelets [33,36]. The described morphology characteristics, i.e., phase coupling degree of composite components, enable/disable charge carries transfer through both materials present within. The band gap values of both composites decreased with SnS_2_ wt % increase. However, this effect became less significant upon exceeding SnS_2_ wt % over 50%, Table 2. Based on the results obtained, the composites possessing SnS_2_ wt % in range 5–50% were set as preferable to be studied in the further tests. Accordingly, the formulation of 72.5 wt % of TiO_2_ and 27.5 wt % of SnS_2_ presents the middle point of such set range.

**Figure 2:**
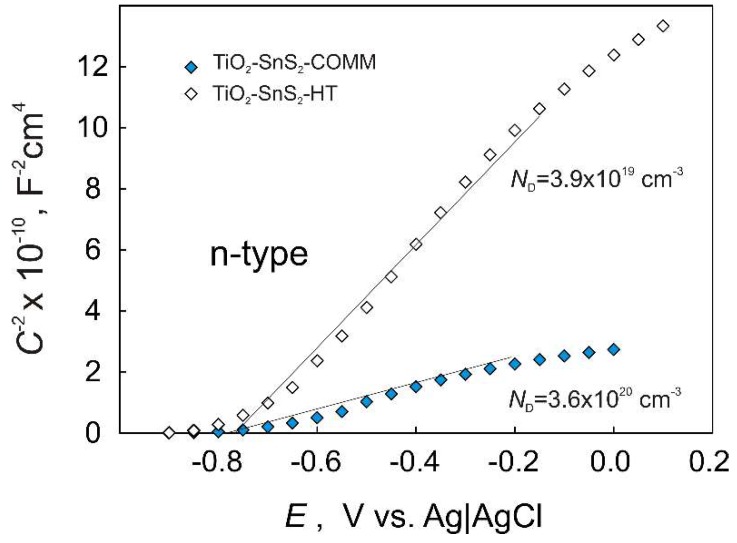
Mott–Schottky plots of the TiO_2_-SnS_2_-COMM and TiO_2_-SnS_2_-HT composite films recorded in NaCl solution.

Semiconducting properties of TiO_2_-SnS_2_-COMM and TiO_2_-SnS_2_-HT films on titanium substrate were further probed by Mott–Schottky analysis. This analysis was performed taking the middle point formulation (27.5 wt % of SnS_2_) for both composites. Mott–Schottky plots for composites (Figure 2) show linear dependence of space charge capacitance ($C_{\mathrm{SC}}^{-2}$) against applied potential (*E*) with a positive slope suggesting n-type semiconducting behavior for both composite films. Under depletion conditions, Mott–Schottky approximation of the capacitance-voltage relationship for n-type semiconductor has the form [37]

(1) $$\;\frac{1}{C_{\mathrm{SC}}^{2}}=\left[\frac{2}{\left(\varepsilon \varepsilon_{0}eN_{D}\right)}\right]\left(E-E_{\mathrm{FB}}-\frac{kT}{e}\right)$$

where *ε*_0_ is vacuum permittivity, *ε* is dielectric constant of composite film, *e* is electron charge, *N*_D_ is donor concentration, *E*_FB_ is flat band potential, *k* is Boltzmann constant, and *T* is thermodynamic temperature. From the slope values (Figure 2), *N*_D_ values equal to 3.6 × 10^20^ cm^−3^ and 3.9 × 10^19^ cm^−3^ were determined for TiO_2_-SnS_2_-COMM and TiO_2_-SnS_2_-HT films, respectively. The lower *N*_D_ values for TiO_2_-SnS_2_-HT in comparison to TiO_2_-SnS_2_-COMM suggests the formation of more crystalline film, as was corroborated by XRD and SEM/EDX results in the previous studies [33,36]. From the slopes and intercepts of Mott–Schottky plots (Figure 2, Equation (1)), *E*_FB_ values equal to −0.81 V and −0.77 V were determined for TiO_2_-SnS_2_-COMM and TiO_2_-SnS_2_-HT films, respectively. The pH of electrolyte solution has strong influence on *E*_FB_ in accordance with Nernstian expression for *E*_FB_ of a semiconductor [38,39]

(2) $$E_{\mathrm{FB}}=-\frac{E_{F}^{0}}{q}+\Delta \phi_{H}$$

(3) $$\Delta \phi_{H}=0.059\;V\cdot ({\mathrm{pH}}_{\mathrm{pzc}}-\mathrm{pH})$$

where pH_pzc_ is the point of zero charge, $E_{F}^{0}$ is Fermi level of semiconductor at pH_pzc_, and $\Delta \phi_{H}$ is the potential drop in Helmholtz double layer at the solid|electrolyte solution interface. At a certain potential, the Fermi level lies at the same energy as the electrolyte solution potential. There is no net transfer of charge, and hence there is no band bending. This potential is therefore referred to as the flat band potential, *E*_FB_.

The specific surface area of studied TiO_2_-SnS_2_ composites and their pure components (TiO_2_ P25, SnS_2_ MKN-900, TiO_2_-HT, and SnS_2_-HT) was determined using BET analysis. The surface charge of studied composites was evaluated by means of their pH_PZC_ values, determined by potentiometric titration. The results are summarized in Table 3 (for composites) and Table S5 (Supplementary Material) (for their pure Components), and presented graphically for pH_PZC_ of both composites (Figure S2, Supplementary material). BET surface areas of studied composites differ significantly (Table 3). Specific area of TiO_2_-SnS_2_-HT is almost twice as large as that of TiO_2_-SnS_2_-COMM. A similar trend in the specific surface area values observed for their pure components was assigned to the particle size and crystalline phase types (Table S5). TiO_2_-HT is characterized by smaller particles comparing to TiO_2_ P25 and exclusively anatase crystalline phase [33,36]. Both SnS_2_ materials possess the same crystalline phase (berndtite), exhibiting rather different morphologies with SnS_2_-HT comprised of significantly smaller structures in comparison to SnS_2_ MKN-900 [33]. Taking into account the pH_PZC_ values determined, it can be concluded that as-synthetized particles have significantly lower pH_PZC_ values than commercial ones (Table 3), which might be reflected in their photocatalytic activity, as investigated further in the study.

**Figure 3:**
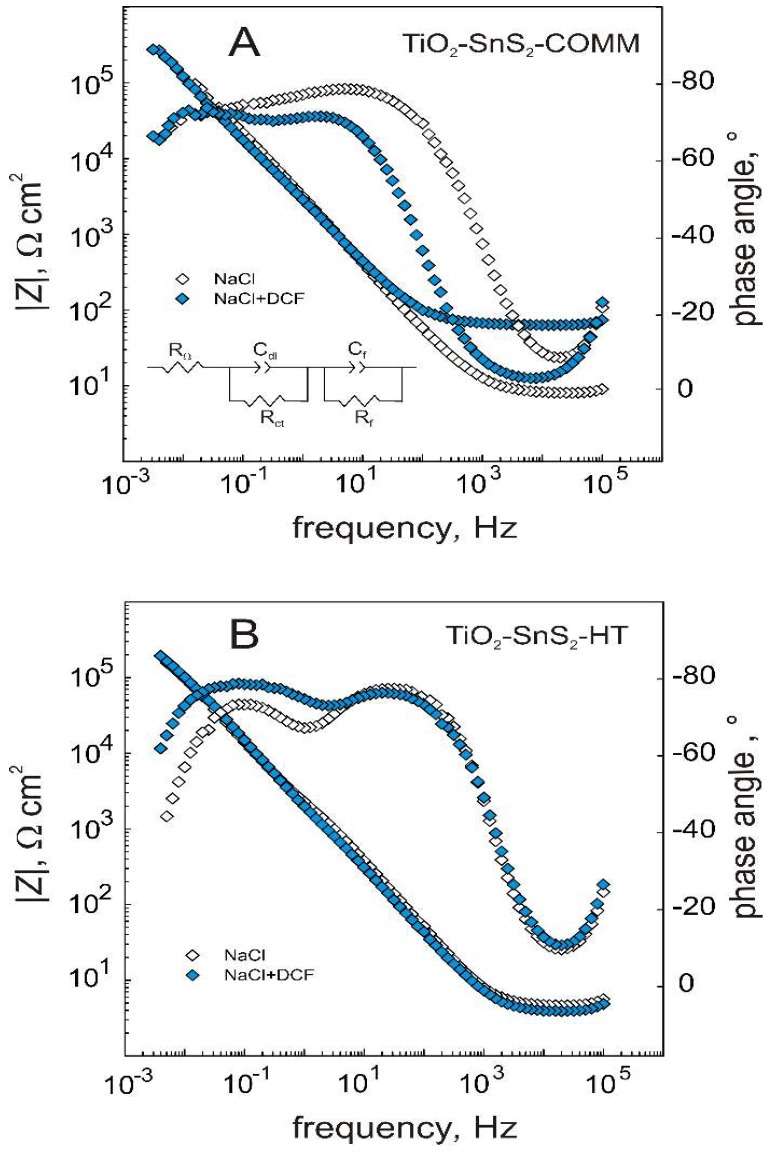
EIS plots of the TiO_2_-SnS_2_-COMM (**A**) and TiO_2_-SnS_2_-HT (**B**) composite films recorded in NaCl and NaCl + DCF solutions (The insert: EEC used to fit the EIS plots given).

The further characterization of composites’ surface properties was conducted by EIS measurements, performed at *E*_ocp_ representing real conditions of potential catalyst application. The impedance spectra of both composite films on Ti plates with and without presence of the organic pollutant DCF (Figure 3), were fitted using EEC (insert in Figure 3); corresponding EEC parameter values are summarized in Table 4. *R*_Ω_ corresponds to the ohmic (electrolyte) resistance. The *R*_ct_ and *C*_dl_ parameters can be linked to the charge transfer processes that occur in an outer porous part of the composite catalyst film at the film–electrolyte interface. Thus, *R*_ct_ is a charge transfer resistance and *C*_dl_ represents the interfacial (double layer) capacitance. The impedance parameters *R*_f_ and *C*_f_ correspond to the resistance and capacitance of the inner part of the barrier composite film. *C*_dl_ of TiO_2_-SnS_2_-HT is several times larger when DCF is present in the electrolyte, indicating a strong and large adsorption extent on the film’s surface. The changes in *C*_f_ and *R*_f_ values are ascribed to different dielectric properties upon formation of the thin adsorption layer. On the other hand, the presence of DCF molecules in the electrolyte influenced *R*_Ω_ and *R*_ct_ values of TiO_2_-SnS_2_-COMM film–electrolyte interface as can be seen in high frequency part of EIS magnitude plots (Figure 3). The impact on the film’s inner part, according to *R*_f_ and *C*_f_ values was negligible also confirming the less degree of DCF adsorption on TiO_2_-SnS_2_-COMM film.

### 3.2. Solar-Driven Photocatalytic Treatment of DCF Using TiO_2_-SnS_2_ Catalysts

**Figure 4:**
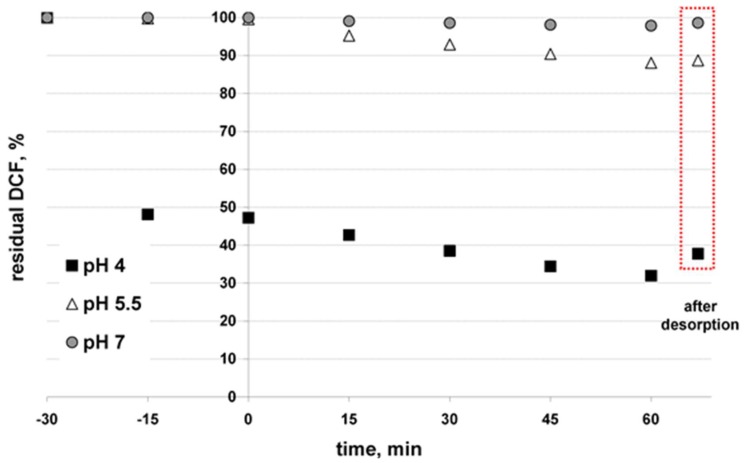
Kinetics of DCF removal by solar/TiO_2_ using reference photocatalytic material; AEROXIDE TiO_2_ P25.

The photoactivity of TiO_2_-SnS_2_-COMM and TiO_2_-SnS_2_-HT composites under simulated solar irradiation was studied using DCF as a model organic pollutant. The influence of key process parameters; initial pH, composite formulation and concentration of H_2_O_2_ oxidant, on the DCF removal/conversion yield was evaluated. The yield results were compared to that obtained using benchmark material, TiO_2_ P25. The determined kinetic profiles of DCF removal by solar/TiO_2_, solar/TiO_2_-SnS_2_-COMM, solar/TiO_2_-SnS_2_-COMM/H_2_O_2_, solar/TiO_2_-SnS_2_-HT, and solar/TiO_2_-SnS_2_-HT/H_2_O_2_ processes are given in Figure 4 and Figures S3–S6 (Supplementary Material), respectively. Two common effects can be observed; (i) the DCF adsorption is closely related to initial pH and (ii) kinetic profiles of DCF removal upon exposure to solar irradiation obeyed first-order kinetics regardless the catalyst material type and formulation, as well as conditions applied. The latter is in good agreement with the literature [40].

**Figure 5:**
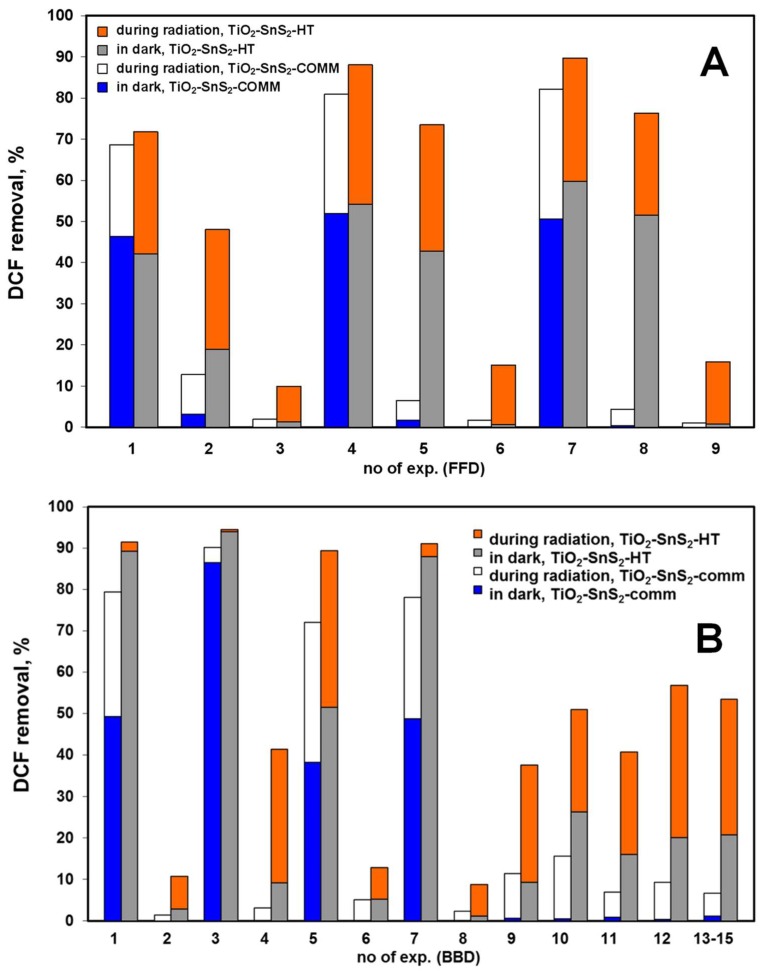
Comparison of DCF removal using TiO_2_-SnS_2_-COMM and TiO_2_-SnS_2_-HT without H_2_O_2_ (**A**) and with H_2_O_2_ addition (**B**) under solar radiation at conditions set by FFD (Table 1, Tables S1 and S3, Supplementary Material) and BBD (Table 1, Tables S2 and S4, Supplementary Material), respectively.

The graphical representations showing only end treatment points, i.e., removals during initial dark period and total, obtained by solar/TiO_2_-SnS_2_-COMM and solar/TiO_2_-SnS_2_-HT (Figure 5A), and solar/TiO_2_-SnS_2_-COMM/H_2_O_2_ and solar/TiO_2_-SnS_2_-HT/H_2_O_2_ processes (Figure 5B), are constructed for experiments conducted according to FFD and BBD (Tables S1–S4, Supplementary Material). The corresponding graphs presenting recorded DCF conversions during the same treatments, performed at the same conditions are given in Figure S7 (Supplementary Material). As can be seen, the adsorption affinity of applied materials, including TiO_2_ P25 (Figure 4), toward DCF in a dark strongly increases by lowering initial pH values. Such effect is related to the surface charge and specific surface area of material, as well as the dissociation constant of DCF. TiO_2_-SnS_2_-COMM has pH_PZC_ value of 6.31, while pH_PZC_ of TiO_2_-SnS_2_-HT is lower (4.61) (Table 3). Furthermore, TiO_2_-SnS_2_-HT has almost double larger specific surface area than TiO_2_-SnS_2_-COMM (Table 2). EIS measurements clearly demonstrated that *C*_dl_ of TiO_2_-SnS_2_-HT is several times larger when DCF is present in the electrolyte (230.6 >> 61.5 μF cm^−2^, Table 4), indicating a strong and large adsorption extent on the film’s surface, which was not the case with TiO_2_-SnS_2_-COMM (63.8 > 45.0 μF cm^−2^). Trovo and Nogueira [41] stated that pKa of DCF is 4.15, meaning that at pH > pKa DCF would be present mostly in its deprotonated form and vice versa. These facts mean that the adsorption of DCF (in its deprotonated form) would be promoted at pH < pH_PZC_ of studied materials. The strongest adsorptions in our case, i.e., DCF removal extents during the initial dark period, were obtained at the lowest studied pH (4) (Figure 5), especially in the case of TiO_2_-SnS_2_-COMM. This material exhibited rather limited adsorption capacity of DCF at other studied pH values (5.5 and 7, Figure 5). Similar results are obtained by benchmark material TiO_2_ P25 (Figure 4), which is built into TiO_2_-SnS_2_-COMM. On the other hand, TiO_2_-SnS_2_-HT showed rather high affinity toward DCF adsorption during initial dark period at pH 5.5 as well (Figure 5). Such results are the consequence of abovementioned differences in BET surface area (Table 2), but also the permeability of immobilized films, as demonstrated by EIS measurements (Figure 3, Table 4). It should be also noted that we have studied the initial pH of DCF solution, thus the immersion of glass plate with immobilized TiO_2_-SnS_2_ composite resulted with the certain decrease of adjusted initial pH toward more acidic pH values (e.g., from 5.5 to 4.65 in the case of TiO_2_-SnS_2_-HT). As can be seen from the results showed in Figure 5, and DCF removal by TiO_2_ P25 (Figure 4), the significant overall DCF removal (≥70%) was accomplished when rather large portion of DCF (≥40%) was adsorbed during initial dark period. Such an effect is particularly pronounced in the case of processes using TiO_2_-SnS_2_-COMM composite. Usually such high removals were followed with rather high DCF conversions as well (Figure 4 and Figure S7, Supplementary Material). The importance of adsorption during dark period and its relationship with high overall removal/conversion upon exposure to solar irradiation can be related with the mechanism of reactive species generation within photocatalytic process. Upon illumination of semiconducting material with the sufficient high energy, *h*ν ≥ *E*_g_, electron and hole pairs (*e*^−^/*h*^+^) formed at catalyst surface enable the generation of radical species or direct oxidation/reduction of adsorbed organics [42]. Hence, effective adsorption of organics during dark period enables their direct degradation at surface either at *e*^−^/*h*^+^ or by formed radicals, overcoming transfer limitations related to their diffusion into the solution where bulk organics can react with them [40,43]. Improved photocatalytic activity of TiO_2_-SnS_2_-HT composite over TiO_2_-SnS_2_-COMM can be attributed to (i) higher number of free charge carriers in TiO_2_-SnS_2_-HT composite (Figure 2) and (ii) lower *R*_ct_ values pointing to facilitated charge transfer across the film (Table 4). A decrease in *R*_ct_ value, when DCF is present, could indicate charge transfer between TiO_2_ and SnS_2_ hetero-junctions mediated by DCF. It is also noteworthy to stress that both composite films possess the excellent corrosion resistance (*R*_corr_), which is a key factor for their application as catalysts in photocatalytic processes for water purification purposes. *R*_corr_ equals to the sum of the resistance components *R*_ct_ and *R*_f_ (Table 4) and for both film is in order of MΩ cm^2^. This result also displays the durability of TiO_2_-SnS_2_ interaction to the inhibition of SnS_2_ leaching and in turn materials’ (catalysts’) photostability. In conclusion, EIS as non-destructive in situ method clearly showed that TiO_2_-SnS_2_-HT composite film exhibits high DCF adsorption degree and offers good electric and charge transfer properties which are key-governing factor for the enhanced efficient photocatalytic activity in the solar driven water treatment. Comparing the effectiveness of processes using composites (Figure 5 and Figure S7, Supplementary Material) to that with benchmark material (Figure 4.), following can be concluded. The adsorption of DCF during initial dark period was similar at the lowest initial pH studied (~50% of DCF was removed), regardless the material used. The increase in SnS_2_ wt % yielded an increase in overall DCF removal and conversion comparing to process using benchmark material, presumably due to higher photoactivity of composites under induced simulated solar irradiation.

**Figure 6:**
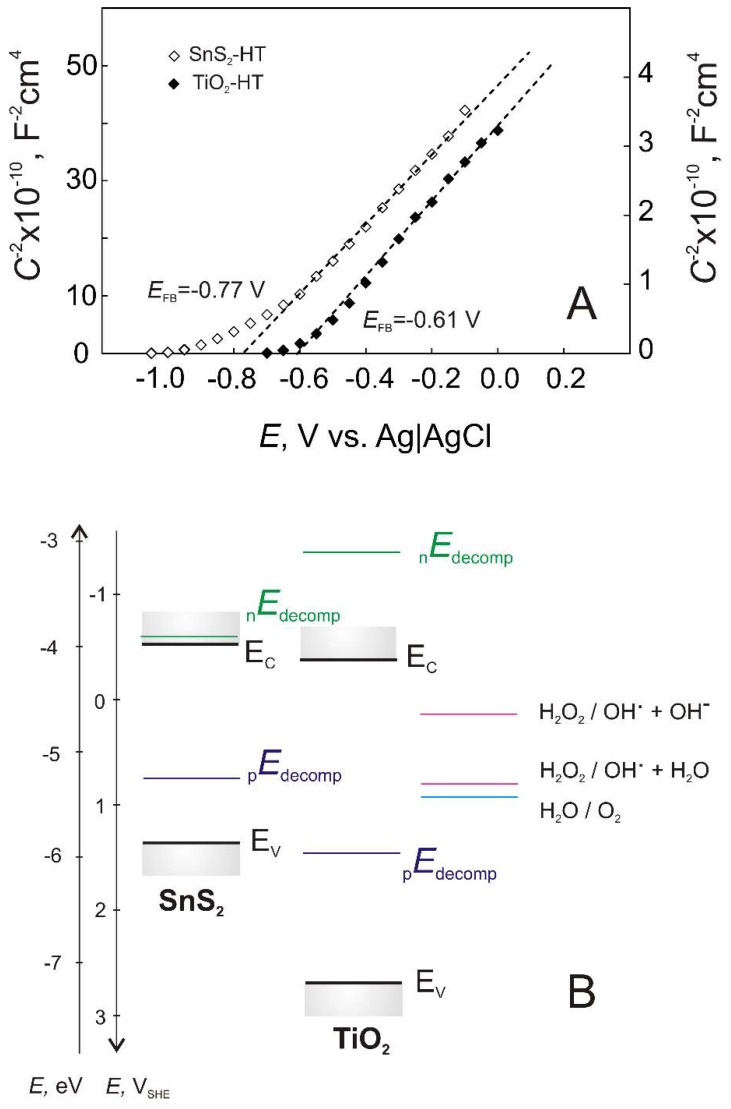
Mott–Schottky plots of the TiO_2_-HT and SnS_2_-HT films recorded in NaCl solution (**A**). The energy band diagram for TiO_2_ and SnS_2_ catalyst films (**B**).

In order to provide deeper insight into effects occurring in TiO_2_-SnS_2_ composites that are responsible for its activity within solar driven water treatment, the semiconducting properties of pure components TiO_2_-HT and SnS_2_-HT were also investigated using Mott–Schottky analysis (Figure 6A). Both catalyst films exhibit positive slope of $C_{\mathrm{SC}}^{-2}$ against *E* dependence, suggesting n-type semiconducting behavior. From the slope and the intercepts values of Mott–Schottky plots (Figure 6A), *N*_D_ and *E*_FB_ were calculated using Equation (1): *N*_D_ values are determined as 1.6 × 10^18^ cm^−3^ and 2.6 × 10^20^ cm^−3^, and *E*_FB_ as −0.61 V and −0.77 for TiO_2_ and SnS_2_, respectively.

Taking into account the semiconducting parameters; *E*_FB_ values and *E*_g_ values (Table 2), the energy band diagram was constructed (Figure 6B). The conduction band (*E*_C_) and valence band (*E*_V_) potentials of SnS_2_ are more negative than those of TiO_2_. This thermodynamically allows the photogenerated electrons to transfer from the SnS_2_ conduction band to TiO_2_ conduction band under visible-light irradiation (λ > 420 nm) that enhances the separation of photogenerated electron and holes in SnS_2_ and bring about the sensitization of TiO_2_. Additionally, the positions of the decomposition potentials of the semiconductor in competition with other redox reactions in the solution were calculated from the thermodynamic data [44,45,46].

According to Gerischer’s approach developed for the binary compound semiconductors [38,47], the reductive decomposition potentials (_n_E_decomp_) of both sulfide film and oxide film lie outside of the band edges, hence both semiconductor components are stable towards theirs reductive decomposition processes, which take place by electrons as major charge carriers in n-type semiconductor

(4) $${\mathrm{TiO}}_{2}+4H^{+}+4e^{-}\to \mathrm{Ti}+2H_{2}O$$

(5) $${\mathrm{SnS}}_{2}+2H^{+}+4e^{-}\to \mathrm{Sn}+2{\mathrm{HS}}^{-}$$

At first glance, the positions of the anodic decomposition potential values (_p_E_decomp_) may suggest the susceptibility of both semiconductor materials to the anodic decomposition; however, when discussing the materials’ stability all competitive redox process at the film–electrolyte interface must be taken into consideration. If redox potentials of solvent are located above _p_E_decomp_ and if these reactions are fast enough, decomposition of the semiconductor would be stabilized in the dark and during solar irradiation conditions [47]. As can be seen from the energy band diagram constructed, based on the redox potential (Fermi level position), the most thermodynamically favorable reaction is the formation of hydroxyl radical, HO• via reaction

(6) $$H_{2}O_{2}+e^{-}\to \mathrm{HO}•+{\mathrm{OH}}^{-}$$

that is desirable for DCF conversion process. The position of calculated energy levels for the anodic decomposition potential (_p_E_decomp_) of SnS_2_

(7) $${\mathrm{SnS}}_{2}+4H_{2}O+2O_{2}{\;+\;8h}_{\mathrm{VB}}^{+}\to {\mathrm{Sn}}^{4+}+2{\mathrm{SO}}_{4}^{2-}+8H^{+}$$

and for reaction

(8) $$H_{2}O_{2}+H^{+}+e^{-}\to \mathrm{HO}•+H_{2}O$$

suggest these are competitive process, thus latter reaction is kinetically more favorable, i.e., formation of HO• is more favorable than SnS_2_ oxidative decomposition. Likewise, the oxidative decomposition of TiO_2_

(9) $${\mathrm{TiO}}_{2}+4{\mathrm{Cl}}^{-}{+\;4h}_{\mathrm{VB}}^{+}\to {\mathrm{TiCl}}_{4}^{}(\mathrm{aq})+O_{2}$$

and O_2_ evolution reaction

(10) $${2H}_{2}{O+\;4h}_{\mathrm{VB}}^{+}\to 4H^{+}+O_{2}$$

are also competitive processes with the latter being thermodynamically and kinetically favorable. In summary, it should be stressed out that even though if conditions of solar irradiation lead to SnS_2_ oxidative decomposition, the composite catalyst material would remain stable and photo-catalytically active due to stable TiO_2_ phase/component since _p_E_decomp_ of TiO_2_ in the presence of chloride ions is below _p_E_decomp_ of SnS_2_. Additionally, reactions (7) and (9) take place using holes as minor charge carriers in the n-type semiconductor.

Since no clear pattern of behavior and influence of studied process parameters (initial pH, composite formulation, and presence and concentration of H_2_O_2_) can be observed neither from the kinetic profiles (Figures S3–S6, Supplementary Material) nor from the end treatment points (providing insight into pH influence only) (Figure 5 and Figure S7, Supplementary Material), the RSM approach was employed. The statistical part on derived RSM models is summarized below. RSM models (M1–M8) were derived (Table S6, Supplementary Material) by applying the multiple regression analysis on FFD or BBD matrices and DCF removal and conversion extents after 60 min treatment (Tables S1–S4, Supplementary Material). The models are characterized for their accuracy, significance, and predictivity by ANOVA (0.982 < *R*^2^ < 0.998 and 0.0001 < *p* < 0.0054; Tables S7–S10, Supplementary Material) and RD tools (example provided in Figure S8, Supplementary Material). According to obtained results, models can be used hereinafter as tools to enlighten the influence of studied parameters of on DCF removal and conversion, correlating determined semiconducting and barrier properties of applied semiconducting materials and occurring mechanisms within the solar driven photocatalytic treatment.

**Figure 7:**
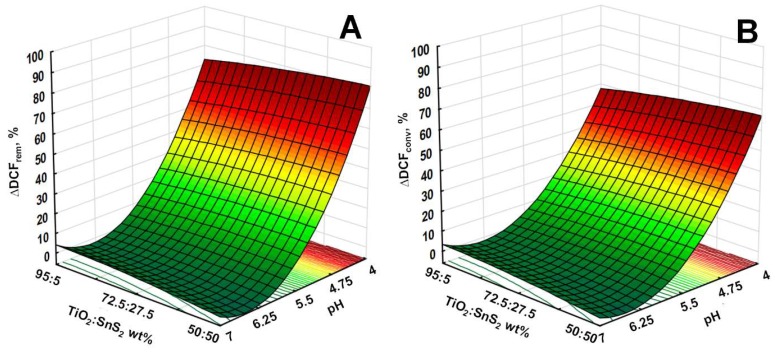
3D response surface and contour diagrams showing the effects of the mutual interactions of initial pH and SnS_2_ wt % on DCF removal (**A**) and conversion (**B**) by solar/TiO_2_-SnS_2_-COMM.

**Figure 8:**
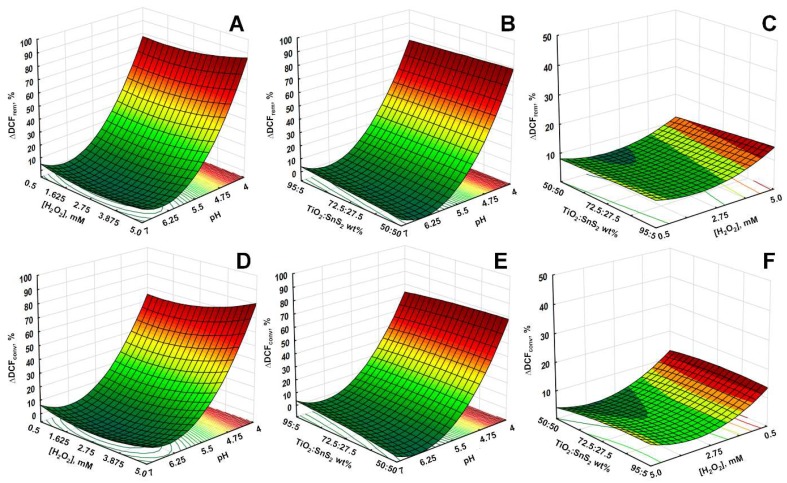
3D response surface and contour diagrams showing the effects of the mutual interactions of initial pH and H_2_O_2_ (**A**,**D**), pH and SnS_2_ wt % (**B**,**E**), and H_2_O_2_ and SnS_2_ wt % (**C**,**F**) on the DCF removal (top row) and conversion (bottom row) by solar/TiO_2_-SnS_2_-COMM/H_2_O_2_ process (SnS_2_ wt %, H_2_O_2_ and pH were held at their respective center levels in (**A**,**D**), (**B**,**E**) and (**C**,F), respectively).

**Figure 9:**
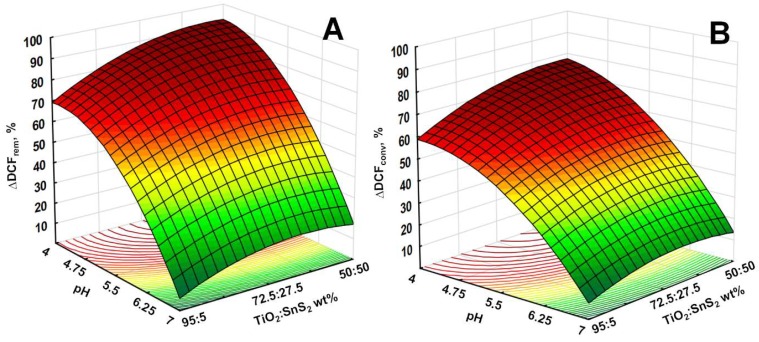
3D response surface and contour diagrams showing the effects of the mutual interactions of initial pH and SnS_2_ wt % on the transformed values of DCF removal (**A**) and conversion (**B**) by solar/TiO_2_-SnS_2_-HT.

**Figure 10:**
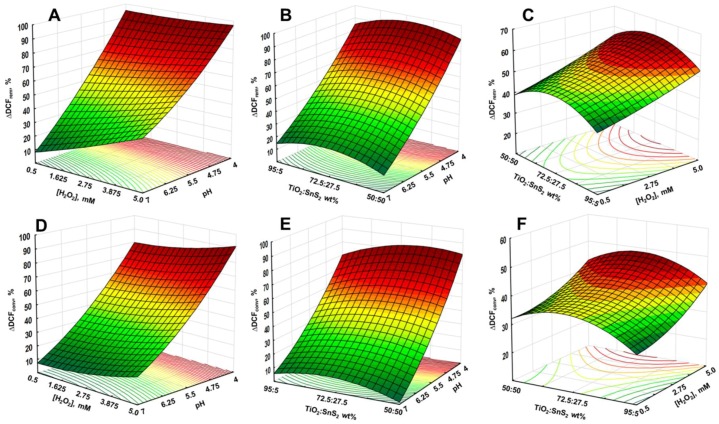
3D response surface and contour diagrams showing the effects of the mutual interactions of initial pH and H_2_O_2_ (**A**,**D**), pH and SnS_2_ wt % (**B**,**E**), and H_2_O_2_ and SnS_2_ wt % (**C**,**F**) on the DCF removal (top row) and conversion (bottom row) by solar/TiO_2_-SnS_2_-HT/H_2_O_2_ process (SnS_2_ wt %, H_2_O_2_ and pH were held at their respective center levels in (**A**,**D**), (**B**,**E**) and (**C**,F), respectively).

The mutual effects of studied process parameters on DCF removal and conversion are presented through 3D surface and contour plots. As discussed above, the pH has significant influence on water treatment by studied processes, particularly on the adsorption during initial dark period and consequently on the overall yield. This effect can be observed from 3D surface and contour plots describing overall DCF removal and conversion by solar/TiO_2_-SnS_2_-COMM (Figure 7), solar/TiO_2_-SnS_2_-COMM/H_2_O_2_ (Figure 8), solar/TiO_2_-SnS_2_-HT (Figure 9), and solar/TiO_2_-SnS_2_-HT/H_2_O_2_ (Figure 10). ANOVA indicated the same trend as well (Tables S7–S10, Supplementary Material). ANOVA also revealed that both H_2_O_2_ (where applicable) and SnS_2_ wt % are significant process parameters. The importance of SnS_2_ wt % in composite can be related to the decrease in band gap values by increasing SnS_2_ content within composite (Table 2). The composites with the lowest SnS_2_ content (5 wt %) exhibited similar effectiveness as TiO_2_ P25 when treatments were conducted in the absence of H_2_O_2_ (Figure 4, Figure 7 and Figure 9). DCF removal and conversion extents obtained at pH 4 were ~70% and ~60%, respectively. However, the increase in SnS_2_ content increased effectiveness of process using TiO_2_-SnS_2_-COMM almost linearly reaching 81% and 67% of DCF removal and conversion, respectively, at pH 4 and 50 wt % of SnS_2_ (Figure 7). TiO_2_-SnS_2_-HT showed even higher effectiveness at comparable conditions (93% and 77% of DCF removal and conversion, respectively) (Figure 9). Its effectiveness increase follows the exponential trend, similar as noticed in the decrease of band gaps values (Table 2). The addition of H_2_O_2_ into the system improved its performance for approximately 10% in both DCF removal and conversion, regardless the composite preparation method (Figure 7, Figure 8, Figure 9 and Figure 10). According to ANOVA (Tables S8 and S10, Supplementary Material) [H_2_O_2_] is a significant process parameter, while the constructed energy–band diagram (Figure 6B) clearly shows favorability of HO• generation through Equations (6) and (8). Due to the fact that no changes in H_2_O_2_ during the treatment were observed (results not showed), one can assume that either (i) H_2_O_2_ was not consumed or (ii) H_2_O_2_ was simultaneously consumed and produced within the system. In the former case, the observed positive effect of H_2_O_2_ (Figure 7, Figure 8, Figure 9 and Figure 10) can be explained by playing a role as a weak acid, thus lowering pH and consequently promoting favorable conditions for the adsorption during the initial dark period. In turn, subsequent conversion at the catalyst surface during the irradiation period is improved as well. However, the latter assumption on simultaneous consumption/generation is more likely to occur as it is supported by the energy band diagram (Figure 6B). The generation of HO• through reactions involving H_2_O_2_ depletion by photogenerated electrons are the most thermodynamically and kinetically favorable anodic reactions (Figure 6B). The optimal wt % of SnS_2_ is also influenced by H_2_O_2_. A lower wt % of SnS_2_ is required to achieve maximum DCF removal and conversion in the presence of H_2_O_2_. In the case of solar/TiO_2_-SnS_2_-HT/H_2_O_2_ 36.1 wt % SnS_2_ was found to be optimal, while 46.5 wt % was deemed optimal for solar/TiO_2_-SnS_2_-HT, according to Figure 9 and Figure 10, respectively. The plausible explanation for such effect can be found in the competitive reactions occurring at anode (7) and (8), as shown through constructed energy band diagram (Figure 6B). Hence, when oxidant is absent, more SnS_2_ would be exposed to decomposition by photogenerated *h*^+^ (7), thus this negative effect should be compensated with higher SnS_2_ content within the composite. On the other hand, SnS_2_ decomposition is suppressed in the presence of H_2_O_2_ due its favorable depletion by photogenerated *e*^−^ (8).

## 4. Conclusions

The TiO_2_-SnS_2_ composite catalysts were prepared by hydrothermal synthesis (TiO_2_-SnS_2_-HT) and by immobilization of the commercial TiO_2_ and SnS_2_ particles (TiO_2_-SnS_2_-COMM) and applied for the solar-driven DCF degradation.

The first property relevant to the photocatalytic activity of TiO_2_-SnS_2_ photocatalyst is its energy band configuration. Energy band diagram was constructed using semiconducting parameters (results of DRS and Mott–Schottky analysis) as well as the thermodynamic data for the determination of the energy levels for the catalysts’ oxidative and reductive decomposition and competitive redox reactions within the band gap. Based on these results, the catalysts’ photocorrosion stability and kinetically preferable redox reactions were discussed in the terms of the solar-driven DCF degradation.

The difference in the conduction band edges of the SnS_2_ and TiO_2_ composite components of approx. 160 mV enabled the transfer of photogenerated electrons from the conduction band of the sensitized narrow-band gap semiconductor SnS_2_ to the conduction band of the wide-band gap TiO_2_ semiconductor. Thus, SnS_2_-TiO_2_ photocatalysts with the heterojunction structure satisfy the light adsorption and charge separation criteria simultaneously, resulting in an efficient photoactivity.

Another key issue influencing the photocatalytic capability of SnS_2_-TiO_2_ photocatalyst is a nature of the surface–interface chemistry. The increased values of the specific area surface (BET analysis) and the adsorption capacity at the solid–solution interfaces (EIS results) and the lower pHpzc values (potentiometric titration) revealed that composite catalyst prepared by HT synthesis shows remarkable improvement in the effectiveness of the DCF removal and conversion during solar-driven water treatment. More SnS_2_-TiO_2_ heterojunctions facilitated the effective photoelectron transfer, which promoted photocatalytic DCF degradation.

Furthermore, thermodynamically favored reactions in the energy band diagrams elucidated the observed effects during the photocatalytic DCF treatment. In the presence of H_2_O_2_ less wt % SnS_2_ in the composite material is required to achieve the highest DCF removal and conversion degree, which is contributed to competitive depletion of H_2_O_2_ by the photogenerated electron. Although the effectiveness of TiO_2_-SnS_2_-COMM is fairly limited at circumneutral conditions, both composites are effective for DCF removal and conversion at acidic pHs and showed higher activity at comparable conditions under solar irradiation than benchmark material TiO_2_ P25. This is particularly valid for TiO_2_-SnS_2_-HT which was more active than TiO_2_ P25 through the entire range of parameters studied.

## Figures and Tables

**Table 1:** Experimental range and levels of the independent variables for the solar driven photocatalytic treatment of DCF using immobilized TiO_2_-SnS_2_-COMM and TiO_2_-SnS_2_-HT in the presence and absence of H_2_O_2_

Process Parameters	Model Variables/Coded Values	Level/Range		
		−1	0	1
pH	*X*_1_	4	5.5	7
H_2_O_2_, mM *	*X*_2_	0.5	2.75	5
SnS_2_, wt % **	*X*_3_	5	27.5	50

* where added. ** in the case of HT composites this is related to the stoichiometric ratio of chemicals in the synthesis.

**Table 2:** Band gap values of TiO_2_-SnS_2_-COMM and TiO_2_-SnS_2_-HT in the dependence to SnS_2_ wt % within composites

TiO_2_-SnS_2_ Catalyst Band Gap; *E*_g_, eV						
Type	SnS_2_ wt %					
	0	5	27.5	50	72.5	100
TiO_2_-SnS_2_-COMM	3.05 *	2.89	2.75	2.67	2.66	1.91
TiO_2_-SnS_2_-HT	3.09	2.99	2.29	2.04	1.96	1.88

* Adopted from previous study [5].

**Table 3:** Surface properties (specific surface area and point of zero charge values) of studied TiO_2_-SnS_2_ composites (72.5:27.5 wt %).

Material	BET Surface Area, m^2^ g^−1^	pH_PZC_
TiO_2_-SnS_2_-COMM	96.00 ± 0.78	6.31
TiO_2_-SnS_2_-HT	160.58 ± 0.46	4.61

**Table 4:** Impedance parameters of TiO_2_-SnS_2_ catalyst films recorded in NaCl and NaCl + DCF solution (*R*_Ω_, *C*_dl_, *R*_ct_, *C*_f_, and *R*_f_ stand for ohmic resistance, double layer capacitance, charge transfer resistance, capacitance and resistance of the composite film, respectively)

**TiO_2_-SnS_2_-COMM**					
Electrolyte	*R*_Ω_, Ω cm^2^	*C*_dl_, μF cm^−2^	*R*_ct_, kΩ cm^2^	*C*_f_, μF cm^−2^	*R*_f_, MΩ cm^2^
NaCl	7.6	45.0	17.63	49.8	1.21
NaCl + DCF	63.3	63.8	4.51	52.3	1.29
**TiO_2_-SnS_2_-HT**					
NaCl	4.5	61.5	0.95	47.8	0.24
NaCl + DCF	4.0	230.6	0.13	31.8	0.81

## Supplementary Materials

The following are available online at http://www.mdpi.com/1996-1944/11/6/1041/s1, Figure S1: Diffuse reflectance spectra of immobilized TiO_2_-SnS_2_ composites with different SnS_2_ wt %; commercial (COMM) (A) and hydrothermal (HT) (B), Figure S2: Determination of pHPZC values TiO_2_-SnS_2_-COMM (A) and TiO_2_-SnS_2_-HT (B) composites, Figure S3: Kinetics of DCF removal by solar/TiO_2_-SnS_2_-COMM process; TiO_2_-SnS_2_-COMM prepared by immobilization using AEROXIDE TIO_2_ P25 and SnS_2_ MKN-900 (Experimental conditions listed in Table 1, and experimental matrix provided by FFD, Table S1, Supplementary material), Figure S4: Kinetics of DCF removal by solar/TiO_2_-SnS_2_-COMM/H_2_O_2_ process; TiO_2_-SnS_2_-COMM prepared by immobilization using AEROXIDE TIO_2_ P25 and SnS_2_ MKN-900 (Experimental conditions listed in Table 1, and experimental matrix provided by BBD, Table S2, Supplementary material), Figure S5: Kinetics of DCF removal by solar/TiO_2_-SnS_2_-HT process; TiO_2_-SnS_2_-HT prepared by hydrothermal method (Experimental conditions listed in Table 1, and experimental matrix provided by FFD, Table S3, Supplementary material), Figure S6: Kinetics of DCF removal by solar/TiO_2_-SnS_2_-HT/H_2_O_2_ process; TiO_2_-SnS_2_-HT prepared by hydrothermal method (Experimental conditions listed in Table 1, and experimental matrix provided by BBD, Table S4, Supplementary material), Figure S7: Comparison of DCF conversion using TiO_2_-SnS_2_-COMM and TiO_2_-SnS_2_-HT without H_2_O_2_ (A) and with H_2_O_2_ addition (B) under solar radiation at conditions set by FFD (Tables 1, and S1 and S3, Supplementary material) and BBD (Tables 1, and S2 and S4, Supplementary material), respectively, Figure S8: Residual diagnostics of model M6 for the prediction of the conversion of DCF by solar/TiO_2_-SnS_2_-HT/H_2_O_2_ process: (A) observed vs. predicted plot, (B) normal probability plot, and (C) internally studentized residuals vs. predicted values plot, Table S1: FFD matrix for removal (M1) and conversion (M2) of diclofenac by solar/TiO_2_-SnS_2_-COMM process after 60 min exposure, Table S2: BBD matrix for removal (M3) and conversion (M4) of diclofenac by solar/TiO_2_-SnS_2_-COMM/H_2_O_2_ process after 60 min exposure, Table S3: FFD matrix for removal (M4) and conversion (M5) of diclofenac by solar/TiO_2_-SnS_2_-HT process after 60 min exposure, Table S4: BBD matrix for removal (M7) and conversion (M8) of diclofenac by solar/TiO_2_-SnS_2_-HT/H_2_O_2_ process after 60 min exposure, Table S5: Specific surface area of constituents of studied TiO_2_-SnS_2_ composites, Table S6: Model equations of derived RSM models for DCF removal and conversion by solar/TiO_2_-SnS_2_ without and with an oxidant H_2_O_2_, Table S7: Analysis of variance (ANOVA) of RSM models M1 and M2 predicting removal and conversion of diclofenac by solar/TiO_2_-SnS_2_-COMM process after 60 min exposure (transformed and non-transformed response values), Table S8: Analysis of variance (ANOVA) of RSM models M3 and M4 predicting removal and conversion of diclofenac by solar/TiO_2_-SnS_2_-COMM/H_2_O_2_ process after 60 min exposure (transformed and non-transformed response values), Table S9: Analysis of variance (ANOVA) of RSM models M5 and M6 predicting removal and conversion of diclofenac by solar/TiO2-SnS2-HT process after 60 min exposure, Table S10: Analysis of variance (ANOVA) of RSM models M7 and M8 predicting removal and conversion of diclofenac by solar/TiO_2_-SnS_2_-HT/H_2_O_2_ process after 60 min exposure.
