# Supplementary material for: Elucidating the Photocatalytic Behavior of TiO2-SnS2 Composites Based on Their Energy Band Structure
Source: Materials (Basel). 2018 Jun 19;11(6):1041. doi: 10.3390/ma11061041 (PMC6024962; doi:10.3390/ma11061041)
Supplement: Supplementary file 1 [file materials-11-01041-s001.pdf]

materials

Supplementary

Elucidating the Photocatalytic Behavior of TiO_2-SnS_2 Composites Based on Their Energy Band Structure

Marin Kovacic, Jozefina Katic, Hrvoje Kusic *, Ana Loncaric Bozic, and Mirjana Metikos Hukovic *

Faculty of Chemical Engineering and Technology, University of Zagreb, Marulicev trg 19, Zagreb 10000, Croatia; mkovacic1@fkit.hr (M.K.); jkatic@fkit.hr (J.K.); abozic@fkit.hr (A.L.B.)

* Correspondence: hkusic@fkit.hr (H.K.); mmetik@fkit.hr (M.M.H.); Tel.: +385-1-4597-123 (H.K.); +385-1-4597-140 (M.M.H.); Fax: +385-1-4597-142 (H.K.); +385-1-4597-139 (M.M.H.)

Received: 29 May 2018; Accepted: 14 June 2018; Published: 19 June 2018

Table S1. FFD matrix for removal (M1) and conversion (M2) of diclofenac by solar/TiO_2-SnS_2-COMM process after 60 min exposure.

	Variables		Experimental results		Response, Y	
Exp. #	X_1	X_3	ΔDCF, %		Y_1	Y_2
	coded	coded	removal	conversion	ΔDCF, % removal	ΔDCF, % conversion
1	-1	-1	68.68	53.89	72.61	57.63
2	0	-1	12.89	11.27	6.84	5.41
3	1	-1	2.01	1.36	4.14	3.47
4	-1	0	80.98	66.06	78.06	63.40
5	0	0	6.42	5.34	8.68	7.40
6	1	0	1.71	1.07	2.38	1.67
7	-1	1	82.18	68.10	81.17	67.02
8	0	1	4.39	3.44	8.19	7.24
9	1	1	1.07	0.44	-1.73	-2.27

Table S2. BBD matrix for removal (M3) and conversion (M4) of diclofenac by solar/TiO_2-SnS_2-COMM/H_2O_2 process after 60 min exposure.

	Variables			Experimental results		Response, Y	
Exp. #	X_1	X_2	X_3	ΔDCF, %		Y_1	Y_2
	coded	coded	coded	removal	conversion	ΔDCF, % removal	ΔDCF, % conversion
1	-1	-1	0	79.41	68.04	77.37	64.93
2	1	-1	0	1.80	1.17	5.26	6.50
3	-1	1	0	90.11	85.78	86.65	80.44
4	1	1	0	3.19	2.93	5.23	6.05
5	-1	0	-1	71.99	56.34	76.50	63.47
6	1	0	-1	5.13	4.92	4.14	3.61
7	-1	0	1	78.05	66.77	79.04	68.08
8	1	0	1	2.37	2.26	-2.13	-4.87
9	0	-1	-1	11.45	11.27	8.98	7.25
10	0	1	-1	15.56	15.31	14.52	13.51
11	0	-1	1	6.99	2.24	8.03	4.04
12	0	1	1	9.27	8.82	11.74	12.84
13	0	0	0	6.64	4.55	6.65	4.55
14	0	0	0	6.64	4.52	6.65	4.55
15	0	0	0	6.66	4.58	6.65	4.55

Table S3. FFD matrix for removal (M4) and conversion (M5) of diclofenac by solar/TiO_2-SnS_2-HT process after 60 min exposure.

Exp. #	Variables		Experimental results		Response, Y	
	X_1	X_3	ΔDCF, %		Y_1	Y_2
	coded	coded	removal	conversion	ΔDCF, % removal	ΔDCF, % conversion
1	-1	-1	71.78	58.26	69.18	58.85
2	0	-1	48.04	45.00	54.95	45.73
3	1	-1	9.98	8.33	5.67	7.01
4	-1	0	88.04	75.35	87.79	73.56
5	0	0	73.47	57.70	70.56	57.44
6	1	0	15.11	13.67	18.28	15.71
7	-1	1	89.71	76.21	92.56	77.41
8	0	1	76.32	58.76	72.33	58.28
9	1	1	15.93	14.27	17.07	13.55

Table S4. BBD matrix for removal (M7) and conversion (M8) of diclofenac by solar/TiO_2-SnS_2-HT/H_2O_2 process after 60 min exposure.

Exp. #	Variables			Experimental results		Response, Y	
	X_1	X_2	X_3	ΔDCF, %		Y_1	Y_2
	coded	coded	coded	removal	conversion	ΔDCF, % removal	ΔDCF, % conversion
1	-1	-1	0	91.50	82.29	95.15	79.54
2	1	-1	0	10.73	7.94	8.15	7.30
3	-1	1	0	94.57	91.02	97.15	91.66
4	1	1	0	41.40	16.52	37.74	19.27
5	-1	0	-1	89.40	69.85	84.86	70.07
6	1	0	-1	12.82	7.57	14.51	5.68
7	-1	0	1	91.02	79.89	89.33	81.78
8	1	0	1	8.73	1.77	13.27	1.54
9	0	-1	-1	37.53	24.54	38.42	27.07
10	0	1	-1	50.98	41.23	52.94	40.36
11	0	-1	1	40.73	31.23	38.77	32.10
12	0	1	1	56.71	45.43	55.82	42.90
13	0	0	0	53.33	39.38	53.47	39.70
14	0	0	0	53.32	39.44	53.47	39.70
15	0	0	0	53.77	40.28	53.47	39.70

Table S5. Specific surface area of constituents of studied TiO_2-SnS_2 composites.

Material	BET surface area, $m^2\ g^{-1}$
TiO_2 P25	50 ± 15 [1]
SnS_2 MKN-900	0.83 ± 0.01
TiO_2-HT	128.39 ± 1.87
SnS_2-HT	22.62 ± 0.29

Table S6. Model equations of derived RSM models for DCF removal and conversion by solar/TiO_2-SnS_2 without and with an oxidant H_2O_2.

Process	Catalyst type	Model #	Model equation
solar/TiO_2-SnS_2	COMM	M1	$Y_1 = 8.68 - 37.84 \times X_1 + 31.54 \times X_1^2 + 0.68 \times X_3 - 1.17 \times X_3^2 - 3.61 \times X_1 \times X_3$

		M2	$Y_2 = 7.40 - 30.86 \times X_1 + 25.14 \times X_1^2 + 0.91 \times X_3 - 1.07 \times X_3^2 - 3.78 \times X_1 \times X_3$
solar/TiO_2-SnS_2/H_2O_2	COMM	M3	$Y_3 = 6.65 - 38.38 \times X_1 + 32.78 \times X_1^2 + 2.31 \times X_2 + 4.21 \times X_2^2 - 0.93 \times X_3 - 0.037 \times X_3^2 - 2.33 \times X_1 \times X_2 - 2.20 \times X_1 \times X_3 - 0.46 \times X_2 \times X_3$
		M4	$Y_4 = 4.55 - 33.20 \times X_1 + 29.05 \times X_1^2 + 3.76 \times X_2 + 5.88 \times X_2^2 - 0.97 \times X_3 - 1.02 \times X_3^2 - 3.99 \times X_1 \times X_2 - 3.27 \times X_1 \times X_3 + 0.64 \times X_2 \times X_3$
solar/TiO_2-SnS_2	HT	M5	$Y_5 = 70.56 -34.75 \times X_1 - 17.52 \times X_1^2 + 8.69 \times X_3 - 6.91 \times X_3^2 - 3.00 \times X_1 \times X_3$
		M6	$Y_6 = 57.44 -28.93 \times X_1 - 12.80 \times X_1^2 + 6.27 \times X_3 - 5.43 \times X_3^2 - 3.00 \times X_1 \times X_3$
solar/TiO_2-SnS_2/H_2O_2	HT	M7	$Y_7 = 53.47 - 36.60 \times X_1 + 5.04 \times X_1^2 + 7.90 \times X_2 + 1.03 \times X_2^2 + 0.81 \times X_3 - 8.02 \times X_3^2 + 6.90 \times X_1 \times X_2 - 1.43 \times X_1 \times X_3 + 0.63 \times X_2 \times X_3$
		M8	$Y_8 = 39.70 - 36.16 \times X_1 + 6.95 \times X_1^2 + 6.02 \times X_2 + 2.79 \times X_2^2 + 1.89 \times X_3 - 6.88 \times X_3^2 - 0.036 \times X_1 \times X_2 - 3.96 \times X_1 \times X_3 - 0.62 \times X_2 \times X_3$

Table S7. Analysis of variance (ANOVA) of RSM models M1 and M2 predicting removal and conversion of diclofenac by solar/TiO_2-SnS_2-COMM process after 60 min exposure (transformed and non-transformed response values).

With non-transformed values

**Factor (coded)**	**Statistical analysis**									
	**SS**		**df**		**MSS**		***F***		***p***	
	**M1**	**M2**	**M1**	**M2**	**M1**	**M2**	**M1**	**M2**	**M1**	**M2**
Model	10638.661	7044.065	5	5	2127.732	1408.813	67.919	48.417	0.0028*	0.0045*
X_1	8591.707	5715.815	1	1	8591.707	5715.815	274.255	196.438	0.0005*	0.0008*
X_1^2	2.737	1263.719	1	1	2.737	1263.719	0.087	43.431	0.7868	0.0071*
X_2	52.105	4.977	1	1	52.105	4.977	1.663	0.171	0.2876	0.7070
X_2^2	1989.379	2.297	1	1	1989.379	2.297	63.503	0.079	0.0041*	0.7970
$X_1 \times X_2$	2.732	57.258	1	1	2.732	57.258	0.087	1.968	0.7870	0.2553
Residual	93.982	87.292	3	3	31.327	29.097				
Total	10732.643	7131.357	8	8						

With transformed values

**Factor (coded)**	**Statistical analysis**									
	**SS**		**df**		**MSS**		***F***		***p***	
	**M1**	**M2**	**M1**	**M2**	**M1**	**M2**	**M1**	**M2**	**M1**	**M2**
Model	0.796	28.833	5	5	0.159	5.767	103.142	67.386	0.0015*	0.0028*
X_1	0.731	27.534	1	1	0.731	27.534	473.293	321.747	0.0002*	0.0004*
X_1^2	0.013	0.091	1	1	0.013	0.091	8.181	1.059	0.0646	0.3791
X_2	0.034	0.725	1	1	0.034	0.725	21.817	8.478	0.0185*	0.0619
X_2^2	0.001	0.015	1	1	0.001	0.015	0.505	0.172	0.5287	0.7058
$X_1 \times X_2$	0.018	0.469	1	1	0.018	0.469	11.915	5.476	0.0409*	0.1012
Residual	0.005	0.257	3	3	0.002	0.086				
Total	0.801	29.090	8	8						

*$p<0.05$ means that model or model term is significant

Table S8. Analysis of variance (ANOVA) of RSM models M3 and M4 predicting removal and conversion of diclofenac by solar/TiO_2-SnS_2-COMM/H_2O_2 process after 60 min exposure (transformed and non-transformed response values).

Factor (coded)	Statistical analysis									
	SS		df		MSS		*F*		*p*	
	M3	M4	M3	M4	M3	M4	M3	M4	M3	M4
**With non-transformed values**										
Model	15881.136	12260.441	9	9	1764.571	1362.271	98.879	30.899	<0.0001*	0.0007*
X_1	11785.644	8820.169	1	1	11785.644	8820.169	660.420	200.058	<0.0001*	<0.0001*
X_1^2	3966.537	3115.435	1	1	3966.537	3115.435	222.269	70.664	<0.0001*	0.0004*
X_2	42.769	113.390	1	1	42.769	113.390	2.397	2.572	0.1823	0.1697
X_2^2	65.323	127.863	1	1	65.323	127.863	3.660	2.900	0.1139	0.1493
X_3	6.956	7.515	1	1	6.956	7.515	0.390	0.170	0.5598	0.6968
X_3^2	0.005	3.854	1	1	0.005	3.854	0.000	0.087	0.9874	0.7794
$X_1 \times X_2$	21.655	63.776	1	1	21.655	63.776	1.213	1.447	0.3208	0.2829
$X_1 \times X_3$	19.409	42.870	1	1	19.409	42.870	1.088	0.972	0.3448	0.3694
$X_2 \times X_3$	0.844	1.618	1	1	0.844	1.618	0.047	0.037	0.8364	0.8556
Residual	89.228	220.440	5	5	17.846	44.088				
Total	15970.365	12480.881	14	14						
**With transformed values**										
**Factor (coded)**	**Statistical analysis**									
	SS		df		MSS		*F*		*p*	
	M3	M4	M3	M4	M3	M4	M3	M4	M3	M4
Model	24.376	27.235	9	9	2.708	3.026	98.253	22.738	<0.0001*	0.0015*
X_1	21.982	21.975	1	1	21.982	21.975	797.443	165.123	<0.0001*	<0.0001*
X_1^2	1.345	2.331	1	1	1.345	2.331	48.806	17.514	0.0009*	0.0086*
X_2	0.208	0.999	1	1	0.208	0.999	7.555	7.510	0.0404*	0.0408*
X_2^2	0.093	0.103	1	1	0.093	0.103	3.362	0.776	0.1262	0.4188
X_3	0.362	0.963	1	1	0.362	0.963	13.138	7.235	0.0151*	0.0433*
X_3^2	0.302	0.456	1	1	0.302	0.456	10.939	3.430	0.0213*	0.1232
$X_1 \times X_2$	0.050	0.118	1	1	0.050	0.118	1.821	0.888	0.2351	0.3893
$X_1 \times X_3$	0.181	0.224	1	1	0.181	0.224	6.570	1.686	0.0505	0.2508
$X_2 \times X_3$	0.000	0.283	1	1	0.000	0.283	0.006	2.130	0.9433	0.2043
Residual	0.138	0.665	5	5	0.028	0.133				
Total	24.513	27.901	14	14						

Table S9. Analysis of variance (ANOVA) of RSM models **M5** and **M6** predicting removal and conversion of diclofenac by solar/TiO_2-SnS_2-HT process after 60 min exposure.

Factor (coded)	Statistical analysis									
	SS		df		MSS		*F*		*p*	
	M5	M6	M5	M6	M5	M6	M5	M6	M5	M6
Model	8444.841	5679.537	5	5	1688.968	1135.907	43.308	279.397	0.0054*	0.0003*
X_1	7245.976	5020.347	1	1	7245.976	5020.347	185.799	1234.846	0.0009*	<0.0001*
X_1^2	614.018	327.830	1	1	614.018	327.830	15.744	80.636	0.0286*	0.0029*
X_2	453.327	236.213	1	1	453.327	236.213	11.624	58.101	0.0422*	0.0047*
X_2^2	95.634	59.073	1	1	95.634	59.073	2.452	14.530	0.2153	0.0317*
$X_1 \times X_2$	35.886	36.074	1	1	35.886	36.074	0.920	8.873	0.4082	0.0587
Residual	116.997	12.197	3	3	38.999	4.066				
Total	8561.837	5691.734	8	8						

*$p<0.05$ means that model or model term is significant

Table S10. Analysis of variance (ANOVA) of RSM models **M7** and **M8** predicting removal and conversion of diclofenac by solar/TiO_2-SnS_2-HT/H_2O_2 process after 60 min exposure .

Factor (coded)	Statistical analysis									
	SS		df		MSS		*F*		*p*	
	M7	M8	M7	M8	M7	M8	M7	M8	M7	M8
Model	11783.882	11252.799	9	9	1309.320	1250.311	67.906	164.620	0.0001*	<0.0001*
X_1	10717.658	10457.913	1	1	10717.66	10457.913	555.855	1376.923	<0.0001*	<0.0001*
X_1^2	93.846	178.300	1	1	93.846	178.300	4.867	23.476	0.0785	0.0047*
X_2	498.655	290.338	1	1	498.655	290.338	25.862	38.227	0.0038*	0.0016*
X_2^2	3.943	28.701	1	1	3.943	28.701	0.205	3.779	0.6700	0.1095
X_3	5.201	28.624	1	1	5.201	28.624	0.270	3.769	0.6257	0.1099
X_3^2	237.463	174.949	1	1	237.463	174.949	12.316	23.034	0.0171*	0.0049*
$X_1 \times X_2$	190.432	0.005	1	1	190.432	0.005	9.876	0.001	0.0256*	0.9801
$X_1 \times X_3$	8.156	62.772	1	1	8.156	62.772	0.423	8.265	0.5441	0.0348*
$X_2 \times X_3$	1.598	1.540	1	1	1.598	1.540	0.083	0.203	0.7850	0.6714
Residual	96.407	37.976	5	5	19.281	7.595				
Total	11880.289	11290.775	14	14						

*p<0.05 means that model or model term is significant

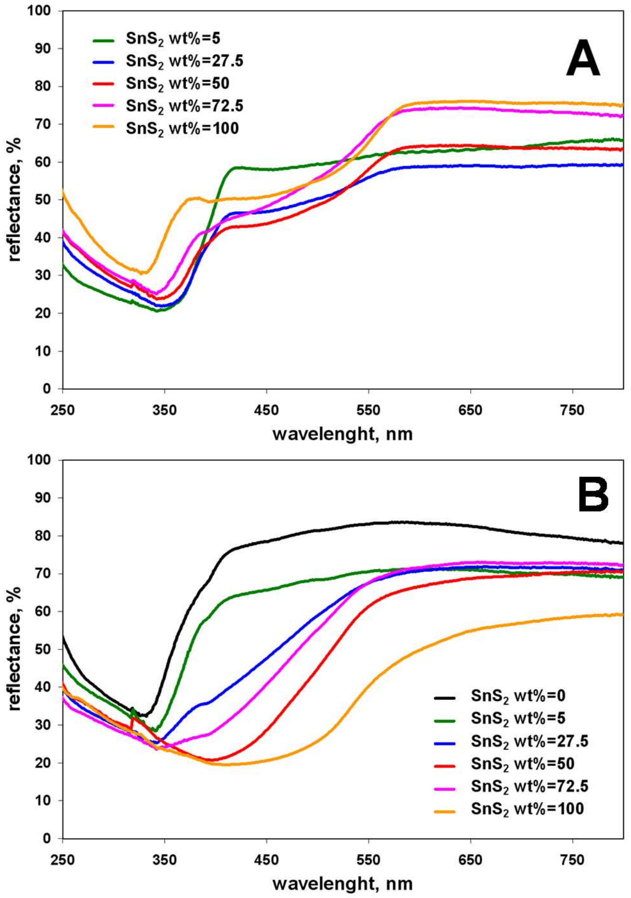

Figure S1. Diffuse reflectance spectra of immobilized TiO_2-SnS_2 composites with different SnS_2 wt%; commercial (COMM) (**A**) and hydrothermal (HT) (**B**)

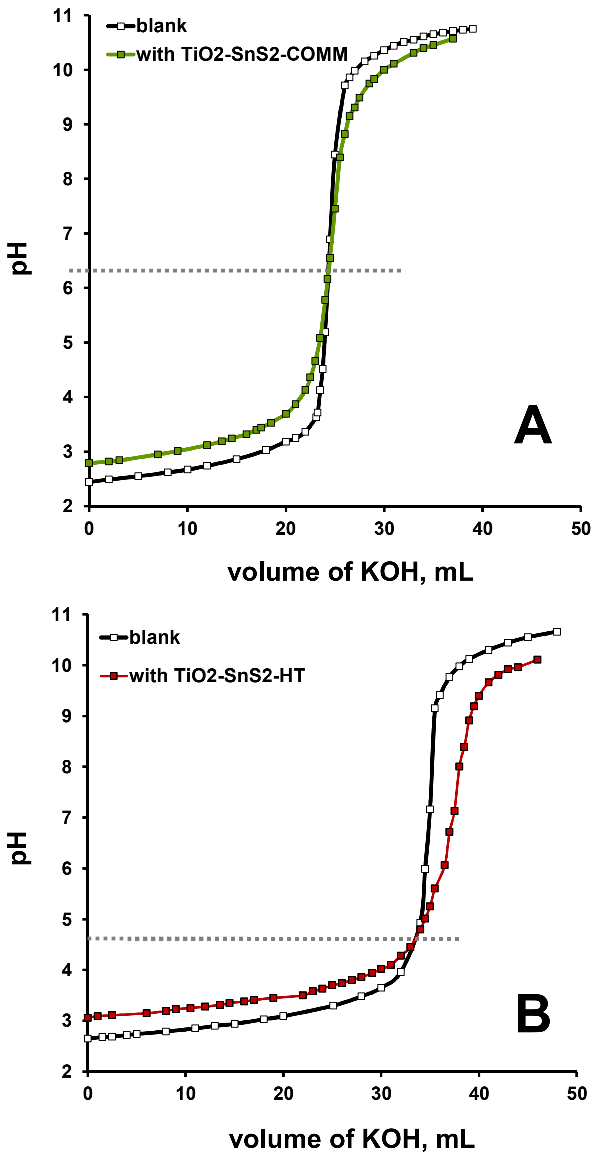

Figure S2. Determination of pH_{PZC} values TiO_2-SnS_2-COMM (**A**) and TiO_2-SnS_2-HT (**B**) composites.

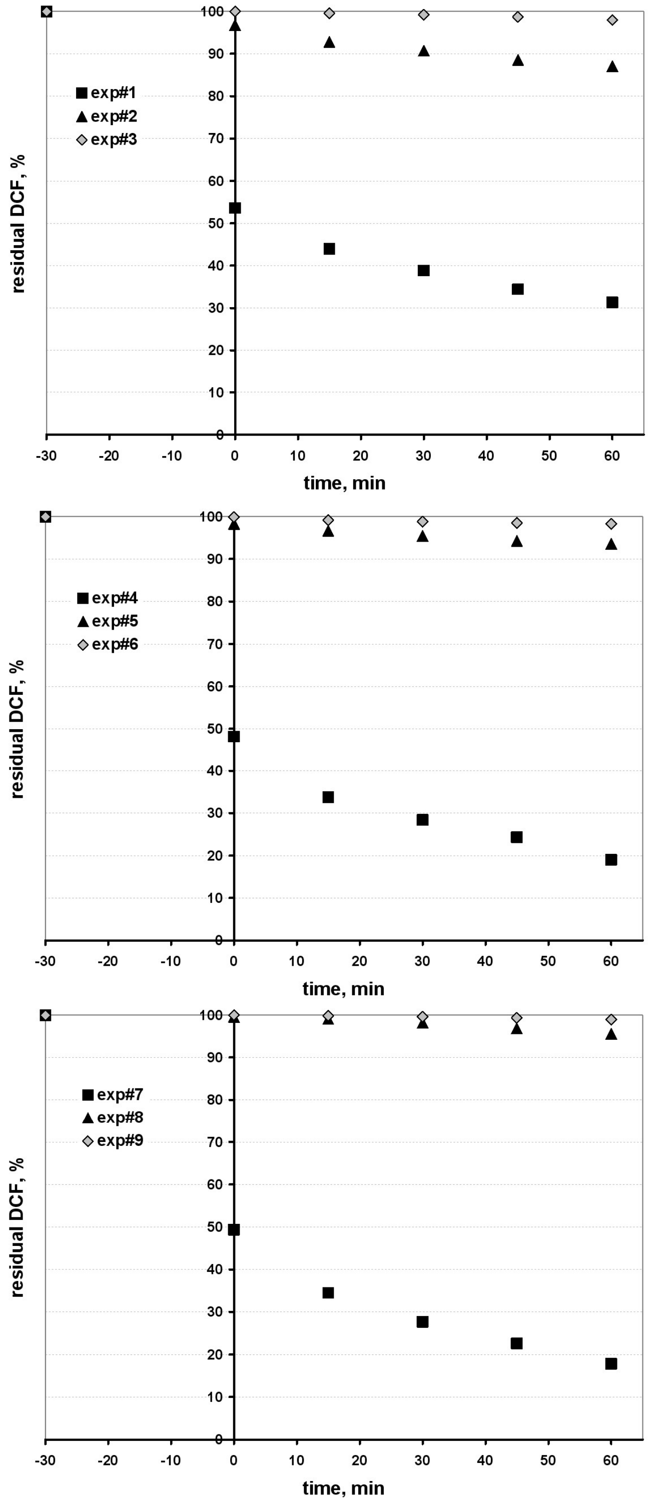

Figure S3. Kinetics of DCF removal by solar/TiO_2-SnS_2-COMM process; TiO_2-SnS_2-COMM prepared by immobilization using AEROXIDE TIO_2 P25 and SnS_2 MKN-900 (Experimental conditions listed in Table 1, and experimental matrix provided by FFD, Table S1, Supplementary material).

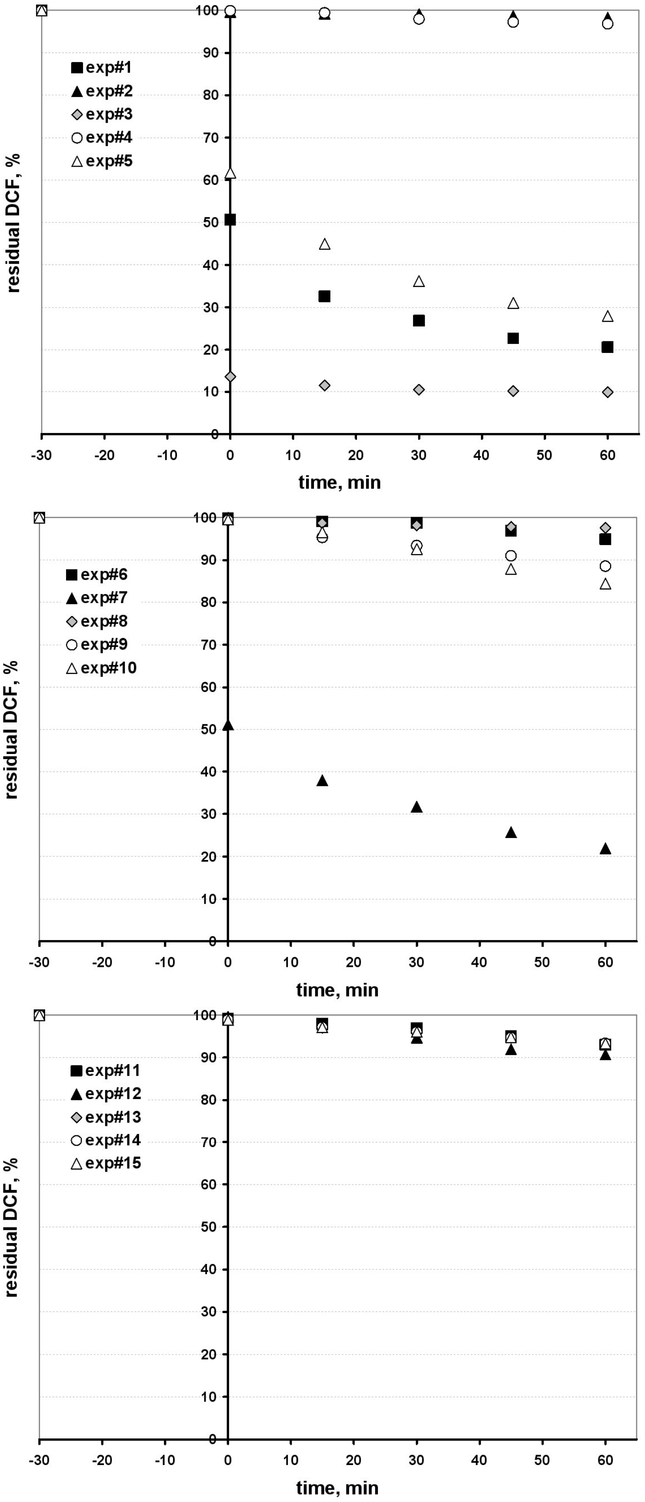

Figure S4. Kinetics of DCF removal by solar/TiO_2-SnS_2-COMM/H_2O_2 process; TiO_2-SnS_2-COMM prepared by immobilization using AEROXIDE TIO_2 P25 and SnS_2 MKN-900 (Experimental conditions listed in Table 1, and experimental matrix provided by BBD, Table S2, Supplementary material).

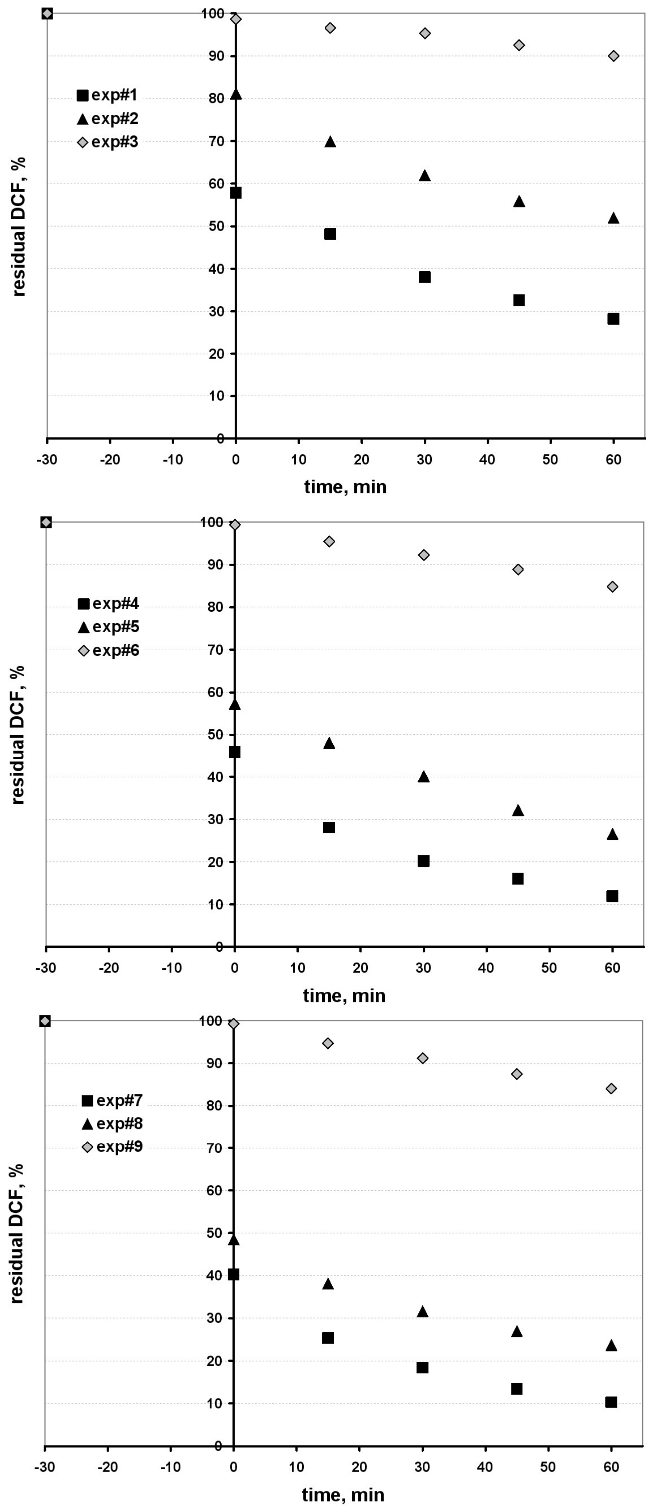

Figure S5. Kinetics of DCF removal by solar/TiO_2-SnS_2-HT process; TiO_2-SnS_2-HT prepared by hydrothermal method (Experimental conditions listed in Table 1, and experimental matrix provided by FFD, Table S3, Supplementary material).

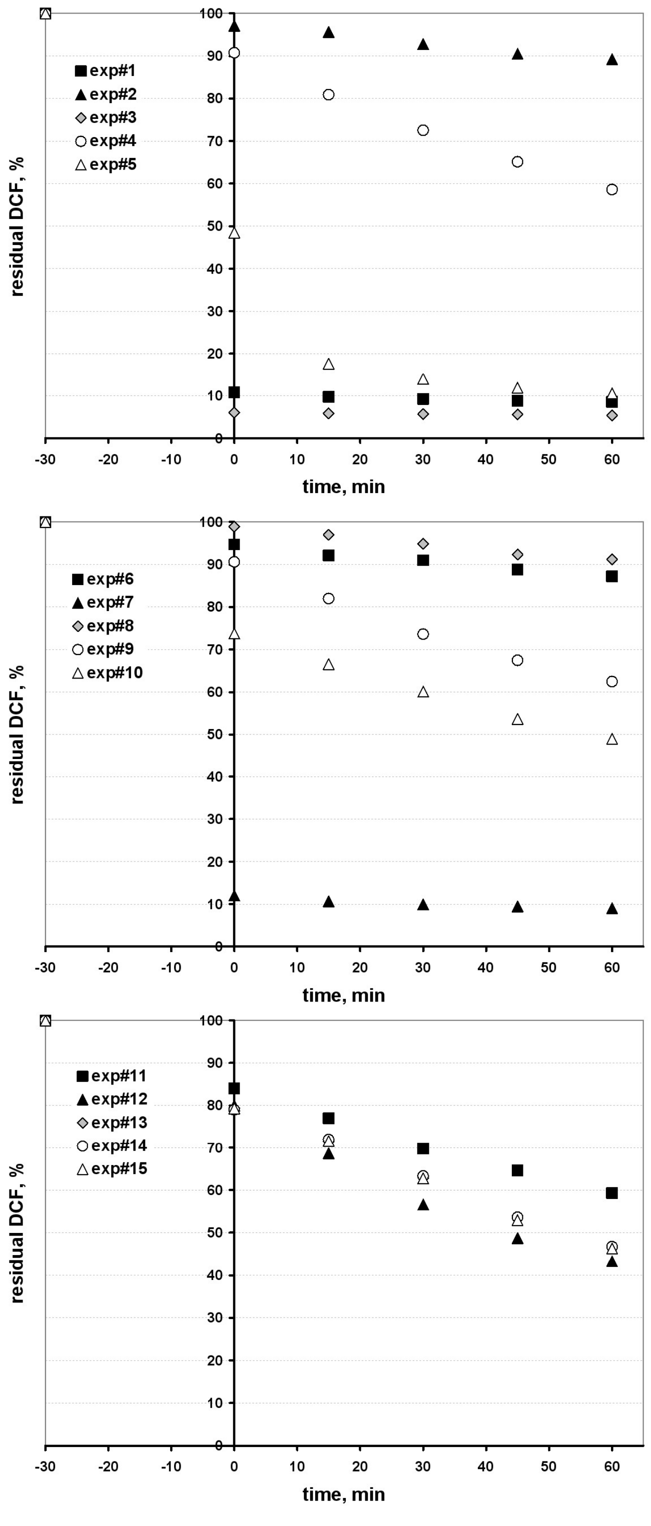

Figure S6. Kinetics of DCF removal by solar/TiO_2-SnS_2-HT/H_2O_2 process; TiO_2-SnS_2-HT prepared by hydrothermal method (Experimental conditions listed in Table 1, and experimental matrix provided by BBD, Table S4, Supplementary material).

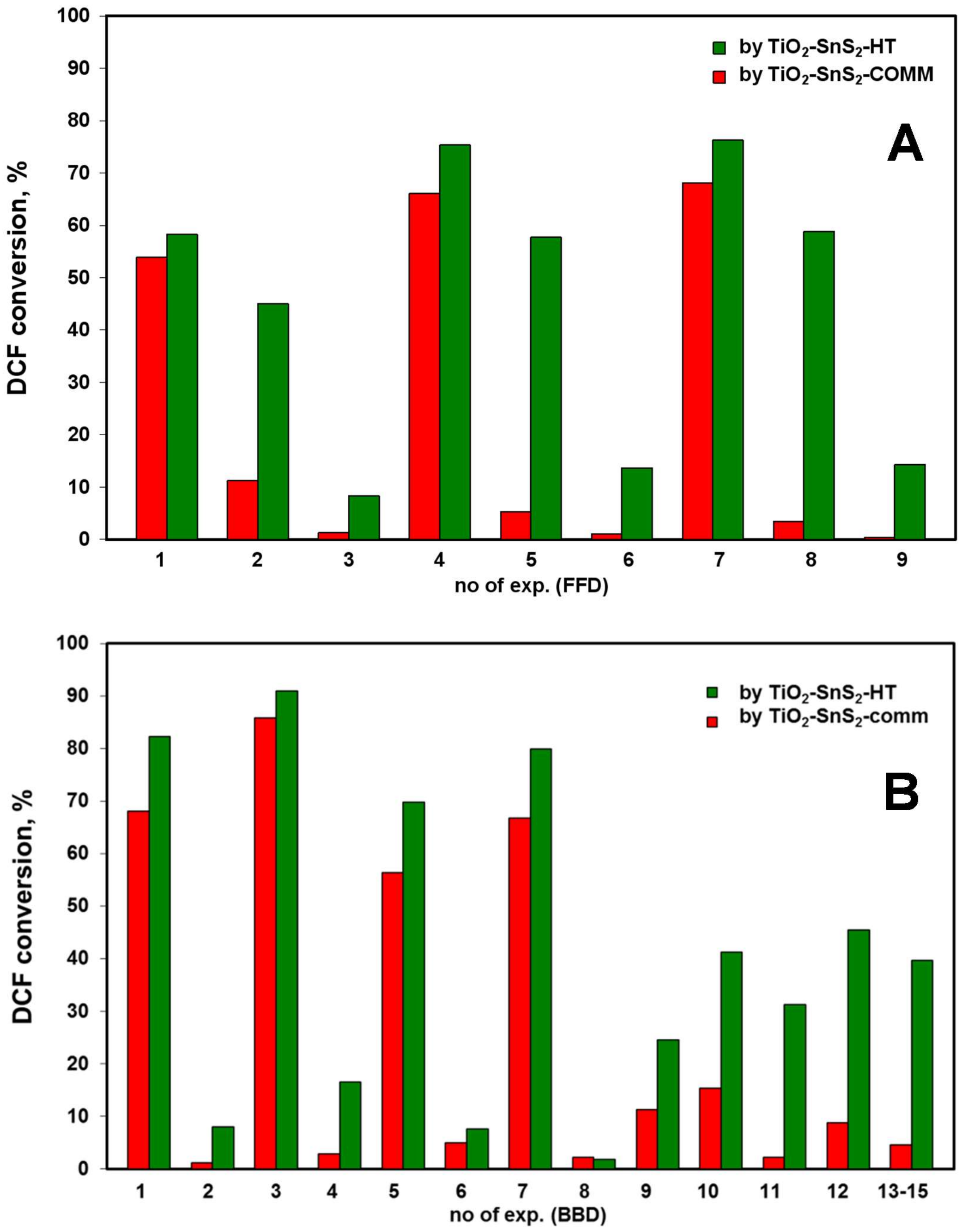

Figure S7. Comparison of DCF conversion using TiO_2-SnS_2-COMM and TiO_2-SnS_2-HT without H_2O_2 (**A**) and with H_2O_2 addition (**B**) under solar radiation at conditions set by FFD (Tables 1, and S1 and S3, Supplementary material) and BBD (Tables 1, and S2 and S4, Supplementary material), respectively.

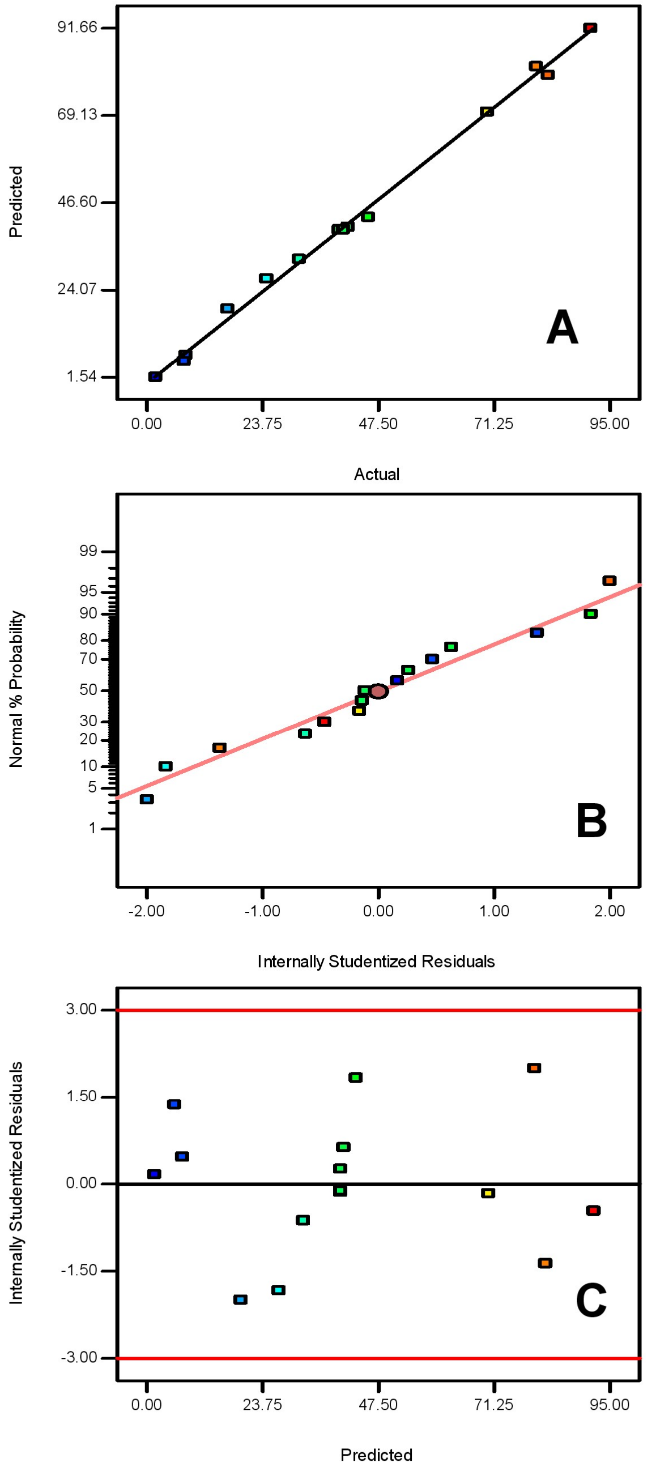

Figure S8. Residual diagnostics of model M6 for the prediction of the conversion of DCF by solar/TiO_2-SnS_2-HT/H_2O_2 process: (**A**) observed *vs.* predicted plot, (**B**) normal probability plot, and (**C**) internally studentized residuals *vs.* predicted values plot.

Detailed Experimental section related to (1) Determination of semiconducting properties by electrochemical measurements, and (2) Calculations and procedure used in RSM modeling

1. .Determination of semiconducting properties by electrochemical measurements

Circular shaped titanium samples (Alfa Aesar, 99.9 wt.% Ti) were abraded with 1000 grit SiC papers, ultrasonically cleaned with ethanol and redistilled water and served as solid substrates for TiO_2-HT and SnS_2-HT pure components, as well as for TiO_2-SnS_2-COMM and TiO_2-SnS_2-HT

composites immobilization as was described in the Experimental section, subsection *2.2. Photocatalysts synthesis and immobilization* (main text). As prepared substrates were embedded in a Teflon holder, with an area, A=1 cm^2 exposed to the solution and were used as working electrodes.

All electrochemical measurements were performed in a conventional three–electrode cell: the working electrode was Ti coated electrode, the counter electrode was a large area platinum electrode and the reference electrode, to which all potentials in the paper are referred, was Ag|AgCl in 3.0 mol dm^{-3} KCl (E = 0.208 V vs. standard hydrogen electrode). The electrolyte was 3% NaCl solution, pure or spiked with DCF (0.1 mM). A Solartron potentiostat/galvanostat 1287 with FRA 1260 controlled by CorrWare® and ZView® softwares was used in these measurements.

The structure of the solid|liquid interface, i.e., the structure of the TiO_2-SnS_2 catalysts|electrolyte solution interface (TiO_2-SnS_2-COMM and TiO_2-SnS_2-HT composite films on titanium substrate) was investigated at the open circuit potential (E_{ocp}) using electrochemical impedance spectroscopy (EIS) performed in the frequency range from 100 kHz to 5 mHz at an *ac* voltage amplitude of ±5 mV. The experimental data were fitted using the complex non–linear least squares (CNLS) fit analysis software [2] and values of the elements of the proposed electric equivalent circuit (EEC) were derived with χ^2 values less than 5 × 10^{-3} (errors in parameter values of 1–3%).

Due to the frequency dispersion (mostly attributed to the "capacitance dispersion"), the capacitor in EECs was replaced with the constant phase element (CPE). The impedance of CPE is defined as $Z(\text{CPE})=[Q(j\omega)^n]^{-1}$, where $j\omega$ is the complex variable for sinusoidal perturbations with $\omega=2\pi f$, and n is the exponent of CPE, while Q is the frequency–independent parameter of CPE, which represents a pure capacitance when n = 1 [3]. Values of 0.70 < n < 1 indicate inhomogeneities at the microscopic level at the metal|electrolyte interface (surface roughness, adsorbed species, etc.) [4,5]. The numerical values of interfacial capacitances, C were calculated using the Brug's relation, valid when the ohmic (electrolyte) resistance, R_Ω is much smaller than the charge–transfer resistance [3]:

$$C = (Q \cdot R_\Omega^{1-n})^{1/n} \quad (1)$$

The electronic–semiconducting properties of TiO_2-HT, SnS_2-HT, TiO_2-SnS_2-COMM and TiO_2-SnS_2-HT catalyst films were investigated by Mott–Schottky method [6]. The capacitance values of the titanium|composite film|solution interface, required for Mott–Schottky analysis, were obtained from EIS measurements. The imaginary part of impedance (Z_{imag}) was recorded as a function of the electrode potential and the frequency (ranging from 3000–30 Hz). The potential was swept in the negative direction from 0 V at a sweep rate of 50 mV s^{-1}. The rapid cathodic scan of 50 mV s^{-1} was used to avoid the change in the film thickness during measurements [7]. From the measured Z_{imag} values, it was possible to calculate CPE parameter $Q = -1/\omega Z_{imag}$ taking into account the angular frequency, $\omega=2\pi f$. From Q value and CPE exponent n and R_Ω, the effective interfacial capacitance, C, was calculated using the expression developed by Brug et al. [3]; eq. (1). The C values consist of the series combination of Helmholtz double layer capacitance (C_H) with the parallel combination of the space–charge capacitance (C_{sc}) and is equal to:

$$C^{-1} = C_H^{-1} + C_{sc}^{-1} \quad (2)$$

All capacitance values were corrected taking Helmholtz capacitance to be 50 μF cm^{-2} [8].

To avoid the frequency dispersion of the effective interfacial capacitance in MS tests and eliminate the contribution of the surface states, the data obtained at seven frequencies (ranging from 3000–30 Hz) were analyzed according to the procedure proposed by Harrington et al. [8,9]. Detailed description of Devine–Harrington procedure can be found in literature [10–12]. By applying Devine–Harrington procedure, the characteristic frequency was determined to be 1000 Hz. Hence the results provided refer to this frequency.

2. .Calculations and procedure used in RSM modeling

The influence of pH, [H_2O_2] and SnS_2 wt % within TiO_2-SnS_2 composites, on DCF removal and conversion was correlated by means of response surface modeling (RSM). The values of process parameters are represented by independent variables: X_1, X_2 and X_3 (Table 1, main text), and according to the number of parameters to be varied within solar driven photocatalytic treatment, experimental matrices were expressed by 3^2 FFD for solar/TiO_2-SnS_2 (Tables S1 and S3, respectively) and BBD for solar/TiO_2-SnS_2/H_2O_2 processes (Tables S2 and S4, respectively). DCF removal and conversion extents after 60 min exposure to solar irradiation were chosen as processes responses (Y). The combined influence of studied parameters on processes performance is described by quadratic polynomial equations, i.e. RSM models [13], and evaluated by the (i) analysis of variance (ANOVA) considering following statistical parameters: Fisher F-test value (F), its probability value (p), regression coefficients (pure; R^2, adjusted; R_{adj}^2, predicted; R_{pre}^2), t-test value, and (ii) graphical based analysis, so-called "residual diagnostic" (RD): including normal probability test, Levene's test, and constant variance test. The calculations were performed by STATISTICA 12.7, StatSoft&Dell; and Design-Expert 10.0, StatEase, software packages.

References

1. Evonik Industries, AEROXIDE®, AERODISP® and AEROPERL® Titanium Dioxide as photocatalyst, Technical information 1243. Available on line: http://www.aerosil.com/sites/lists/RE/DocumentsSI/TI-1243-Titanium-Dioxide-as-Photocatalyst-EN.pdf. (Accessed on May 02, 2018)
2. Boukamp, A. A nonlinear least squares fit procedure for analysis of immittance data of electrochemical systems, Solid State Ion. **1986**, *20*, 31–44.
3. Brug, G.J.; Van der Eeden, A.L.G.; Sluyters-Rehbach, M.; Sluyters, J.H. The analysis of electrode impedances complicated by the presence of a constant phase element. *J. Electroanal. Chem*. 1984, 176 275–295.
4. Jorcin, J.B.; Orazem, M.E.; Pebere, N.; Tribollet, B. CPE analysis by local electrochemical impedance spectroscopy. *Electrochim. Acta* **2006**, *51*, 1473–1479.
5. Lasia, A. Electrochemical Impedance Spectroscopy and its Applications, in *Modern Aspects of Electrochemistry*; Conway, B.E.; Bockris, J.; White, R.E.; Eds.; Kluwer Academic/Plenum Publishers: New York, NY, USA, 1999; Volume 32, p. 143–248.
6. Orazem, M.E.; Tribollet, B. *Electrochemical Impedance Spectroscopy*, John Wiley & Sons: New York, NY, USA, 2008.
7. Harrington, S.P.; Devine, T.M.; Analysis of electrodes displaying frequency dispersion in Mott-Shottky tests. *J. Electrochem. Soc*. **2008**, *155*, C381–C386.
8. Katić, J.; Metikoš-Huković, M.; Šarić, I.; Petravić, M. Semiconducting properties of the oxide films formed on tin: Capacitive and XPS studies. *J. Electrochem. Soc*. **2016**, *163*, C221–C227.
9. Harrington, S.P.; Wang, F.; Devine, T.M. The structure and electronic properties of passive and prepassive films of iron in borate buffer. *Electrochim. Acta* **2010**, *55*, 4092–4102.
10. Katić, J.; Metikoš-Huković, M. Correlation between electronic and corrosion properties of the passive oxide film on Nitinol. *Acta Chim. Slov*. **2014**, *61*, 350–356.
11. Katić, J.; Metikoš-Huković, M.; Milošev, I. Ionic and electronic conductivity of the anodic films on nickel. *J. Electrochem. Soc*. **2015**, *162*, C767–C774.
12. Katić, J.; Metikoš-Huković, M.; Šarić, I.; Petravić, M. Electronic structure and redox behavior of tin sulfide films potentiostatically formed on tin. *J. Electrochem. Soc.* **2017**, *164*, C383–C389.
13. Myers, R.H.; Montgomery, D.C.; Anderson-Cook, C.M. *Response Surface Methodology: Process and Product Optimization Using Designed Experiments*, 3rd ed.; John Wiley & Sons: Hoboken, NJ, USA, 2009.

© 2018 by the authors. Submitted for possible open access publication under the terms and conditions of the Creative Commons Attribution (CC BY) license (http://creativecommons.org/licenses/by/4.0/).
